# Incidence and risk factors of acute kidney injury in cancer patients treated with immune checkpoint inhibitors: a systematic review and meta-analysis

**DOI:** 10.3389/fimmu.2023.1173952

**Published:** 2023-05-29

**Authors:** Caihong Liu, Wei Wei, Letian Yang, Jian Li, Cheng Yi, Yajun Pu, Ting Yin, Feifei Na, Ling Zhang, Ping Fu, Yuliang Zhao

**Affiliations:** ^1^ Department of Nephrology, Kidney Research Institute, West China Hospital of Sichuan University, Chengdu, China; ^2^ Department of Thyroid and Parathyroid Surgery, West China Hospital, Sichuan University, Chengdu, Sichuan, China; ^3^ Department of Thoracic Oncology, Cancer Center, and State Key Laboratory of Biotherapy, West China Hospital, Sichuan University, Chengdu, China

**Keywords:** immune checkpoint inhibitors, acute kidney injury, cancer, incidence, risk factors

## Abstract

**Background:**

The incidence and risk factors of acute kidney injury (AKI) in patients with malignancies receiving immune checkpoint inhibitors (ICIs) are being extensively reported with their widespread application.

**Objective:**

This study aimed to quantify the incidence and identify risk factors of AKI in cancer patients treated with ICIs.

**Methods:**

We searched the electronic databases of PubMed/Medline, Web of Science, Cochrane and Embase before 1 February 2023 on the incidence and risk factors of AKI in patients receiving ICIs and registered the protocol in PROSPERO (CRD42023391939). A random-effect meta-analysis was performed to quantify the pooled incidence estimate of AKI, identify risk factors with pooled odds ratios (ORs) and 95% confidence intervals (95% CIs) and investigate the median latency period of ICI-AKI in patients treated with ICIs. Assessment of study quality, meta-regression, and sensitivity and publication bias analyses were conducted.

**Results:**

In total, 27 studies consisting of 24048 participants were included in this systematic review and meta-analysis. The overall pooled incidence of AKI secondary to ICIs was 5.7% (95% CI: 3.7%-8.2%). Significant risk factors were older age (OR: 1.01, 95% CI: 1.00–1.03), preexisting chronic kidney disease (CKD) (OR: 2.90, 95% CI: 1.65–5.11), ipilimumab (OR: 2.66, 95% CI: 1.42–4.98), combination of ICIs (OR: 2.45, 95% CI: 1.40–4.31), extrarenal immune-related adverse events (irAEs) (OR: 2.34, 95% CI: 1.53-3.59), and proton pump inhibitor (PPI) (OR: 2.23, 95% CI: 1.88–2.64), nonsteroidal anti-inflammatory drug (NSAID) (OR: 2.61, 95% CI: 1.90–3.57), fluindione (OR: 6.48, 95% CI: 2.72–15.46), diuretic (OR: 1.78, 95% CI: 1.32–2.40) and angiotensin-converting enzyme inhibitors (ACEIs) or angiotensin-receptor blockers (ARBs) (pooled OR: 1.76, 95% CI: 1.15–2.68) use. Median time from ICIs initiation to AKI was 108.07 days. Sensitivity and publication bias analyses indicated robust results for this study.

**Conclusion:**

The occurrence of AKI following ICIs was not uncommon, with an incidence of 5.7% and a median time interval of 108.07 days after ICIs initiation. Older age, preexisting chronic kidney disease (CKD), ipilimumab, combined use of ICIs, extrarenal irAEs, and PPI, NSAID, fluindione, diuretics and ACEI/ARB use are risk factors for AKI in patients receiving ICIs.

**Systematic review registration:**

https://www.crd.york.ac.uk/prospero/, identifier CRD42023391939.

## 1 Introduction

Cancer is the leading cause of death worldwide (1). Oncologists urgently need to develop novel remedies that effectively target tumor cells to break new ground on this universal conundrum. The human immune system is the superior force in killing cancer cells, whose activation can be inhibited by the combination of two transmembrane proteins located in T cells known as immune checkpoints, cytotoxic T lymphocyte-associated antigen 4 (CTLA-4) and programmed cell death protein 1 (PD-1), and their corresponding ligand proteins B7, PD-L1/PD-L2, respectively (2, 3). Tumor cells cunningly take advantage of this negative regulation beneficial for immune tolerance to evade the body’s immune attack (4). Immune checkpoint inhibitors (ICIs), as monoclonal antibodies of immunotherapy to interrupt the binding of CTLA-4 and B7, PD-1 and PD-L1/PD-L2, have revolutionized the treatment paradigm of a variety of advanced malignancies with surprising results, especially melanoma and lung cancer (5).

Along with the wide application of ICIs in cancer treatment, multisystem autoimmune phenomena termed immune-related adverse events (irAEs) have markedly emerged into our vision for their nonselective role on immune checkpoints. The overall incidences of irAEs in patients treated with ICIs range from 15% to 90%, of which the most frequently observed are dermal, gastrointestinal tract, endocrine system and liver (6, 7). Although renal-associated irAEs are relatively rare, fatal complications such as acute kidney injury (AKI) should be seriously considered. In total, the use of ICIs was linked to an increased risk of all-grade AKI (RR = 1.37, 95% CI: 1.14–1.65), as indicated by a pharmacovigilance study (8). However, high inconsistency of the incidence of AKI after ICI use, ranging from 0. 4% to 28.1%, brings about a challenge for clinicians to determine evidence-based therapy strategies (9, 10). Simultaneously, a growing body of studies has pioneered efforts to investigate risk factors of AKI occurring with ICIs. The first study performed by Seethapathy et al. in 2019 found that exposure to PPIs was associated with a significantly increased risk for sustained ICI-AKI (11). Both similar and opposite results were detected in a series of later works but lacked consensus (12, 13). In addition, differential diagnoses and renal biopsy of ICI-AKI are challenging in clinical practice. In light of this, we regard median onset time AKI after ICIs initiation as a valuable implication for clinicians. Knowledge of the overall incidence and risk factors of AKI following ICIs, involving sex, older age, comorbidities, concomitant agents and so on, is controversial and not comprehensive to date. To address this gap, this systematic review and meta-analysis is aimed at providing an updated result of the quantified incidence and risk factors of AKI in patients treated with ICIs, as well as median time from ICIs initiation to the occurrence of AKI.

## 2 Methods

### 2.1 Protocol and guidance

The study protocol has already been registered in PROSPERO (CRD42023391939) and in accordance with the Cochrane handbook and Preferred Reporting Items for Systematic Reviews and Meta-Analyses (PRISMA) (14).

### 2.2 Search strategy

Electronic databases of English language publications, PubMed/Medline, Web of Science, Cochrane and Embase were searched to collect studies dated to 1 February 2023 in terms of the incidence and risk factors of AKI in patients treated with ICIs. We retrieved relevant articles through the combination of Medical Subject Headings (MeSH) terms comprising “immune checkpoint inhibitors”, “acute kidney injury”, “risk factor” or “incidence” and all the corresponding free words. Complete and detailed search strategies of each database are provided in **Supplementary Appendix S1** for incidence and **Supplementary Appendix S2** for risk factors. Searches were limited to English but with no date, sex, or ethnicity restrictions.

### 2.3 Inclusion and exclusion criteria

The inclusion criteria were as follows: (a) the study sample consisted of patients with malignancy and treated with ICIs aged 18 years or above; (b) the study reported the incidence or risk factors of AKI in patients receiving ICIs. AKI is defined as an increase in serum creatinine to≥1.5 times the baseline level, which is known or presumed to have occurred within the prior 7 days; or an increase in serum creatinine by ≥26.5 µmol/L within 48 hours; or oliguria (urine volume <0.5 ml/kg per hour for 6 hours; and (c) sufficient data were provided on the event number or effect size, the odds ratios (OR) or hazard ratio (HR), and 95% confidence intervals (95% CI) with full texts.

Studies were excluded for the following reasons: (a) they were reviews, meta-analyses, case reports, conference abstracts and guidelines; and (b) the study was conducted based on animals.

### 2.4 Study selection

**Figure 1:**
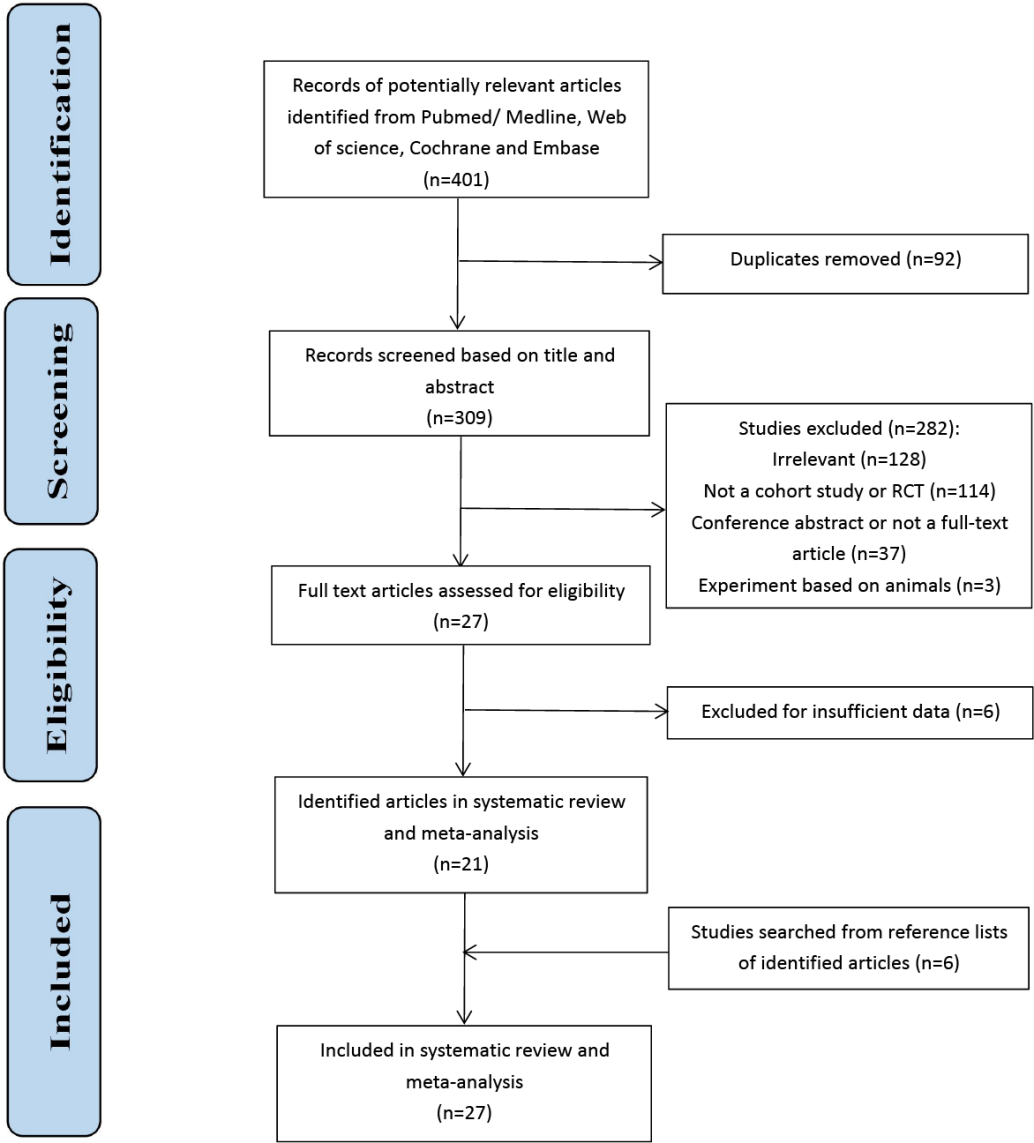
Flowchart of the selection process.

After excluding duplicates, two researchers independently screened the titles and abstracts of all identified records to remove irrelevant documents. Then, a full-text review was conducted to determine the eligibility for inclusion. Any disagreement on study selection was resolved by discussion with the third researcher (LC, ZY, and WW). The study selection process is shown in **Figure 1**.

### 2.5 Data extraction and quality assessment

The following data were extracted from the included studies: (a) the basic information, including first author, publication year, region, data source, data, study design, study type, and enrollment period; (b) characteristics of the participants, including sample size, number of AKI cases, sex ratio (male), and median age; and (c) the incidence, median time and interquartile ranges and effect size (OR or HR) and 95% CI of potential risk factors in each study, encompassing sex, older age, comorbidities, baseline eGFR, ICIs, extrarenal irAEs, and concomitant agents. We prioritized the extraction of multivariate analysis data from the adjustment model. According to the Newcastle−Ottawa Scale (NOS) designed for cohort studies and case−control studies and the Cochrane Handbook designed for randomized clinical trial, two researchers (LC and ZY) independently assessed the risk of bias. For 23 observational studies, selection (0-4 scores), comparability (0-2 scores) and outcome or exposure (0-3 scores) are the three evaluation domains containing an eight-item checklist ranging from 0 to 9 scores. The scores of the included articles ranged from 6 to 9 and were classified as high quality. Among the three domains, the outcome of the included records performed the worst due to scant time for the occurrence of potential ICI-AKI. For 4 randomized clinical trials, 2 studies that did not adopt blinding methods for participants and outcome were evaluated as ‘‘High risk.” A study failing to elaborate specific method of allocation concealment was classified as ‘‘Unclear”. Other evaluation items were mostly ‘‘Low risk.” Generally, the included studies had low methodological bias. Detailed information is illustrated in **Supplementary Table S1** and **Supplementary Figures S1**, **S2**. Any disagreement was discussed with another reviewer (LC, ZY and WW).

### 2.6 Statistical analysis

In this meta-analysis, the incidence, median time from ICIs initiation and risk factors of AKI encompassing sex, older age, comorbidities, baseline eGFR, ICIs, extrarenal irAEs, and concomitant agents in cancer patients receiving ICIs were pooled and compared between patients with AKI and without AKI using random effects and *I* ^2^ statistics for between-study heterogeneity (*I* ^2^>50%, *P*<0.05 implied potential heterogeneity). Meta-regression was conducted to explore the contributor of substantial heterogeneity. OR and 95% CI were employed as pooled effects, and *P*<0.05 was considered to be statistically significant. We regarded HR as a good estimate of OR only if HR was represented in original studies. IBM SPSS Statistics 20 was used to calculate ORs and 95% CIs if neither ORs nor HRs were provided in the primary texts. All statistical analyses were performed using Stata Statistical Software, version 16.0. Moreover, sensitivity analyses by sequentially omitting studies and publication bias analyses by funnel plots, Begg’s and Egger’s statistical tests were shown to evaluate the robustness of the results.

## 3 Results

### 3.1 Eligible studies

A total of 401 documents were initially searched for incidence and risk factors. After removing 92 duplicates, we screened the title and abstract of each study and found that 128 citations were apparently irrelevant. In the remaining records, 3 studies based on animals, 15 meta-analyses, 80 reviews, 19 identified as case reports and guidelines were deleted. Besides, 19 conference abstracts and 18 without full articles were also excluded. Then, 27 full texts were scrutinized, and 6 studies were excluded for insufficient data on risk factors. In addition, 6 studies were added by searching from reference lists of 21 identified articles. Finally, 27 literatures were carried out in this systematic review and meta-analysis, with 22 for incidence, 17 for risk factors and 12 shared (9–13, 15–36).

### 3.2 Study characteristics

A total of 24048 participants receiving different types of ICIs due to malignancies from 27 studies were investigated in our systematic review and meta-analysis. The sample size varied between 89 and 3109, and the median age of recorded patients ranged from a minimum of 57 years to a maximum of 69 years. The majority were native to America and Asia. These articles were all published between 2017 and 2022, and studies were performed from 2010 to 2021. Despite 4 randomized clinical trials and a case-control study, the remaining 22 were cohort studies (**Table 1**).

**Table 1:** Characteristics of the studies included in the meta-analysis.

First author	Year	Region	Data source	Data	Study design	Study type	Sex, Male%	Sample size	AKI	Median age	Enrollment period	ES	Adjusted
Abdelrahim	2021	America	monocenter	incidence, risk factors	retrospective	cohort study	66%	1664	58	63 years	2010.1-2019.11	hazard ratio	adjusted
Antonia	2017	America	multicenter	incidence	double-blind	randomized clinical trial	70.10%	713	2	64 years	2014.5-2016.4	NA	NA
Bellmunt	2021	America	multicenter	incidence	open-label	randomized clinical trial	78.86%	809	3	NA	2015.10-2018.7	NA	NA
Cortazar	2020	United States and Canada	multicenter	risk factors	retrospective	cohort study	NA	414	138	NA	NA	odds ratio	adjusted
De Giglio	2022	Italy	monocenter	incidence, risk factors	retrospective	cohort study	60.70%	89	25	69 years	2020.3-2021.11	NA	crude
Espi	2021	France	monocenter	incidence, risk factors	retrospective	cohort study	63.35%	352	13	67 years	2015.1-2017.7	odds ratio	adjusted
Gandhi	2018	America	multicenter	incidence	double-blind	randomized clinical trial	58.93%	616	22	65 years	2016.2-2017.3	NA	NA
Garcia-Carro	2022	Palestine	monocenter	risk factors	retrospective	cohort study	59%	759	118	64 years	2018.3-2019.5	odds ratio	adjusted
Gerard	2022	France	multicenter	risk factors	retrospective	case–control study	20.70%	845	167	NA	NA	odds ratio	adjusted
Gupta	2021	North America, Europe, and Asia	multicenter	risk factors	retrospective	cohort study	60%	858	429	NA	2012 to 2020	odds ratio	adjusted
Gupta	2022	America	monocenter	incidence, risk factors	NA	cohort study	50.92%	872	52	67.5 years	NA	hazard ratio	adjusted
Isik	2021	America	monocenter	incidence	retrospective	cohort study	50%	2143	37	NA	2014.1-2020.6	NA	NA
Ji	2022	China	monocenter	incidence, risk factors	retrospective	cohort study	69%	1615	114	57.41 years	2014.1-2019.12	odds ratio	adjusted
Koks	2021	Netherlands	monocenter	incidence, risk factors	retrospective	cohort study	62.10%	676	96	64 years	2013.1-2020.5	hazard ratio	adjusted
Liu	2022	China	monocenter	incidence, risk factors	retrospective	cohort study	80.60%	305	14	64 years	2018.1-2020.8	odds ratio	adjusted
Meraz-Muñoz	2020	Canada	monocenter	incidence, risk factors	retrospective	cohort study	60%	309	51	61 years	2010.1-2017.1	odds ratio	adjusted
Powles	2020	England	multicenter	incidence	open-label	randomized clinical trial	75.48%	1032	6	NA	2015.11-2017.3	NA	NA
Qin	2022	China	monocenter	incidence, risk factors	retrospective	cohort study	69.50%	551	31	62 years	2017.12-2020.1	NA	adjusted
Seethapathy	2019	America	monocenter	incidence, risk factors	retrospective	cohort study	61%	1016	30	63 years	2011.5-2016.12	hazard ratio	adjusted
Seethapathy	2020	America	monocenter	incidence	retrospective	cohort study	50%	599	5	65 years	2017.1-2018.12	NA	NA
Seethapathy	2021	America	multicenter	incidence	retrospective	cohort study	NA	637	159	NA	2012-2018	NA	NA
Shimamura	2021	Japan	monocenter	incidence, risk factors	retrospective	cohort study	75%	152	27	67 years	2015.3-2019.10	odds ratio	adjusted
Sorah	2021	America	monocenter	incidence	retrospective	cohort study	NA	1766	14	NA	2014.4-2018.12	NA	NA
Stein	2021	France	monocenter	incidence, risk factors	retrospective	cohort study	55%	239	41	66.2 years	2014.1-2018.2	hazard ratio	adjusted
Strohbehn	2021	America	multicenter	risk factors	retrospective	cohort study	43.50%	3109	NA	62.8 years	2010-2019	hazard ratio	adjusted
Trevisani	2022	Italy	multicenter	incidence	retrospective	cohort study	67.10%	292	26	66 years	2017.1-2020.1	NA	NA
Yu	2022	China	monocenter	incidence	retrospective	cohort study	63.20%	1616	68	57 years	2014.1-2019.12	NA	NA

AKI, acute kidney injury; ES, effect size. NA, not available.

### 3.3 The incidence of AKI following ICIs

**Figure 2:**
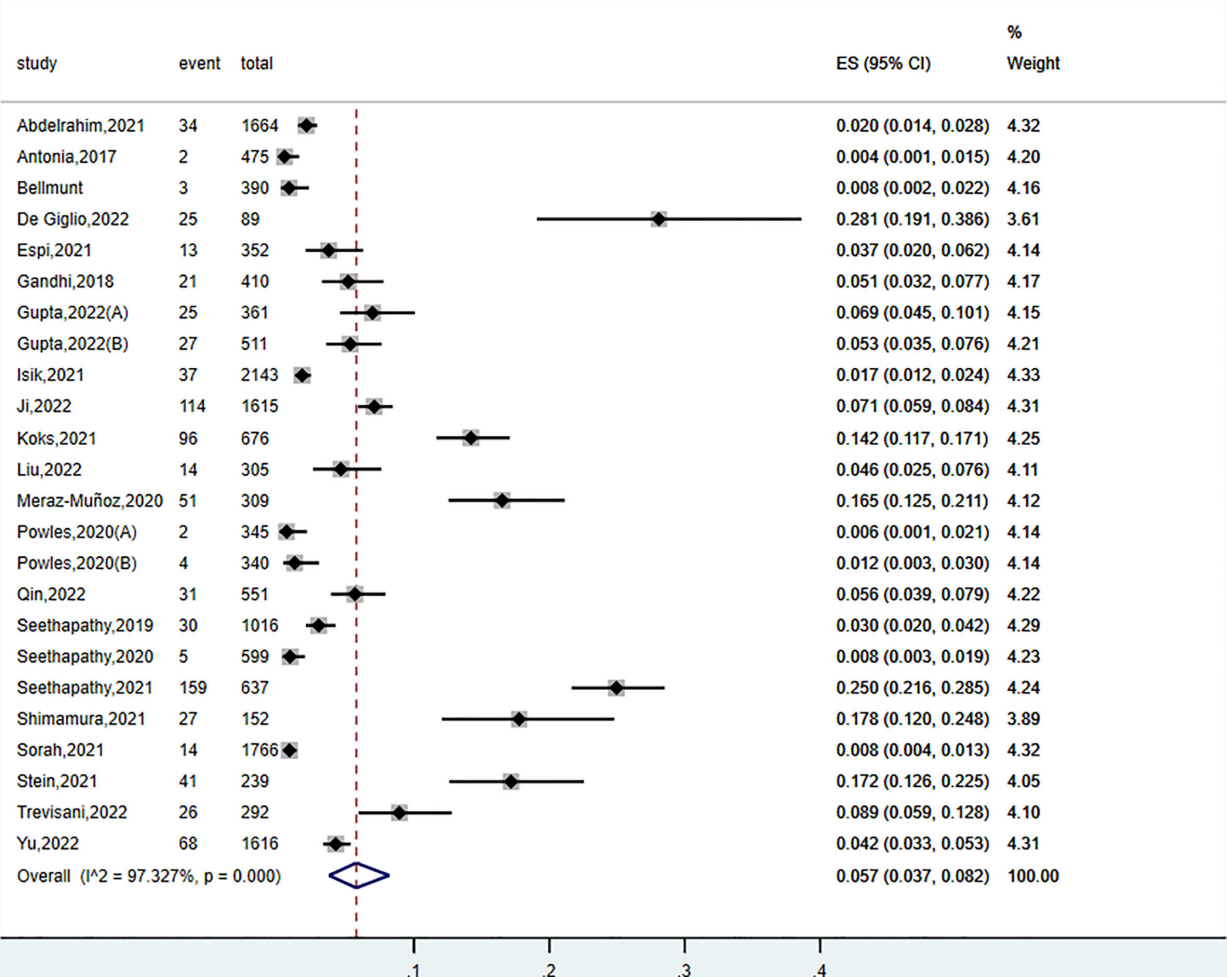
Forest plots of the incidence of AKI in patients treated with ICIs.

The incidence of AKI secondary to ICIs in patients with malignancies was available from 22 articles. Morbidity ranging from 0.4% to 28.1% was relatively low compared with other irAEs, such as colitis and rash (37). The overall pooled incidence of AKI following ICIs was 5.7% (95% CI: 3.7%-8.2%, *P*< 0.001, *I* ^2^ = 97.3%) (**Figure 2**). Meta-regression was performed to explore the source of noted heterogeneity. We analyzed seven covariates, including region, publication year, sample size, follow-up time, male ratio, malignancy type, and ICI type. Nevertheless, none of them was acknowledged as a potential contributor to heterogeneity.

### 3.4 Risk factors for AKI following ICIs

**Figure 3:**
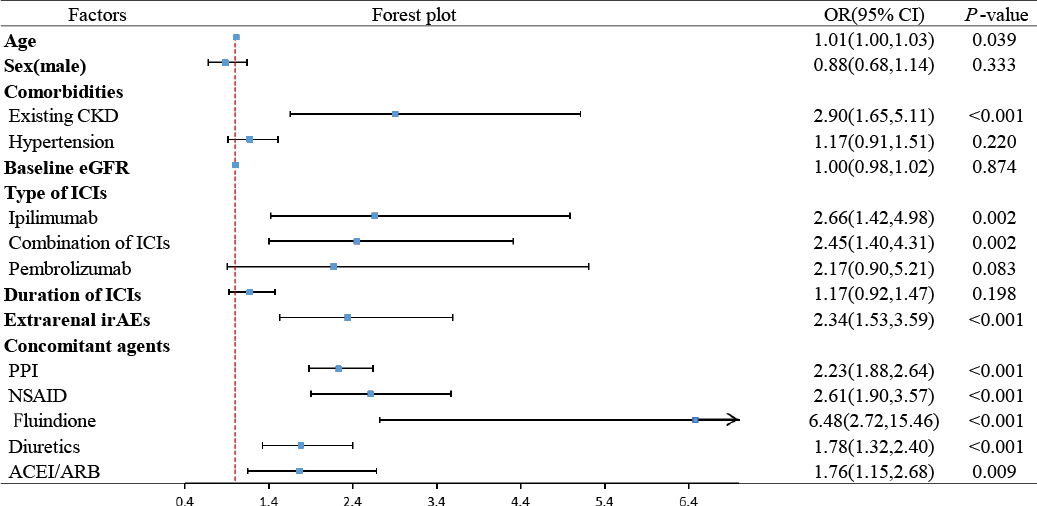
Forest plot of summary results of risk factors for AKI in patients treated with ICIs.

Data on risk factors for AKI in patients suffering from cancer during ICI treatment were reported by 17 papers. In most articles, comparisons were made between participants in the AKI group and the no AKI group. The summary statistics of potential risk factors are shown in **Table 2** and **Figure 3**.

**Table 2:** Meta-analysis of risk factors for AKI in patients treated with ICIs: summary of results.

Factors	No. study	Heterogeneity test		OR (95% CI)	*P* value
		*P* value	*I* ^2^(%)		
**Age**	9	0.453	0	1.01 (1.00,1.03)	0.039
**Sex (male)**	10	0.001	67.2	0.88 (0.68,1.14)	0.333
Comorbidities					
Preexisting CKD	2	0.511	0	2.90 (1.65,5.11)	< 0.001
Hypertension	6	0.499	0	1.17 (0.91,1.51)	0.220
**Baseline eGFR**	5	<0.001	85.5	1.00 (0.98,1.02)	0.874
Type of ICIs					
Ipilimumab	2	0.592	0	2.66 (1.42,4.98)	0.002
Combination of ICIs	6	0.003	72.3	2.45 (1.40,4.31)	0.002
Pembrolizumab	2	0.114	60.0	2.17 (0.90,5.21)	0.083
**Duration of ICIs**	3	0.003	83.2	1.17 (0.92,1.47)	0.198
**Extrarenal irAEs**	6	0.002	74.4	2.34 (1.53,3.59)	< 0.001
Concomitant agents					
PPI	8	0.811	0	2.23 (1.88,2.64)	< 0.001
NSAID	5	0.652	0	2.61 (1.90,3.57)	< 0.001
Fluindione	2	0.983	0	6.48 (2.72,15.46)	< 0.001
Diuretics	5	0.781	0	1.78 (1.32,2.40)	< 0.001
ACEI/ARB	6	0.103	45.5	1.76 (1.15,2.68)	0.009

AKI, acute kidney injury; ICIs, immune checkpoint inhibitors; OR, odds ratio; 95% CI, 95% confidence intervals; PPI, proton pump inhibitors; NSAID, nonsteroidal anti-inflammatory drugs; ACEI, angiotensin-converting enzyme inhibitor; ARB, angiotensin-receptor blocker; CKD, chronic kidney disease; irAEs, immune-related adverse events; eGFR, estimated glomerular filtration rate.

#### 3.4.1 Age, sex and comorbidities

**Figure 4:**
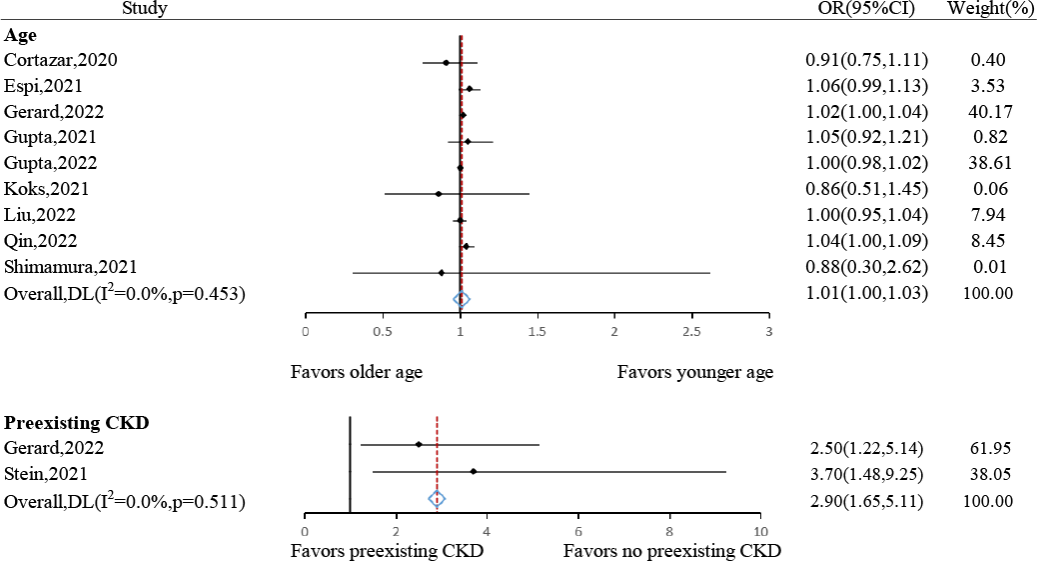
Forest plots of the odds ratio for age and preexisting CKD.

The risk of AKI following ICIs generally increased with advancing age (pooled OR: 1.01, 95% CI: 1.00–1.03, *P*= 0.039, *I* ^2^ = 0.0%, n= 9 studies) (**Figure 4**). However, sex gap was not proven to be associated with AKI (male: pooled OR: 0.88, 95% CI: 0.68-1.14, *P*= 0.333, *I* ^2^ = 67.2%, n= 10 studies) (**Supplementary Figure S3**)

The risk of contracting AKI after ICIs was significantly higher in patients with preexisting CKD (pooled OR: 2.90, 95% CI: 1.65–5.11, *P*< 0.001, *I* ^2^ = 0.0%, n= 2 studies) (**Figure 4**), but not in those with hypertension (pooled OR: 1.17, 95% CI: 0.91–1.51, *P*= 0.220, *I* ^2^ = 0.0%, n= 6 studies) (**Supplementary Figure S4**). Regarding eGFR, no statistically significant relationship with AKI was showed by our results (pooled OR: 1.00, 95% CI: 0.98–1.02, *P*= 0.874, *I* ^2^ = 85.5%, n= 5 studies) (**Supplementary Figure S5**).

#### 3.4.2 Type of ICIs and extrarenal irAEs

**Figure 5:**
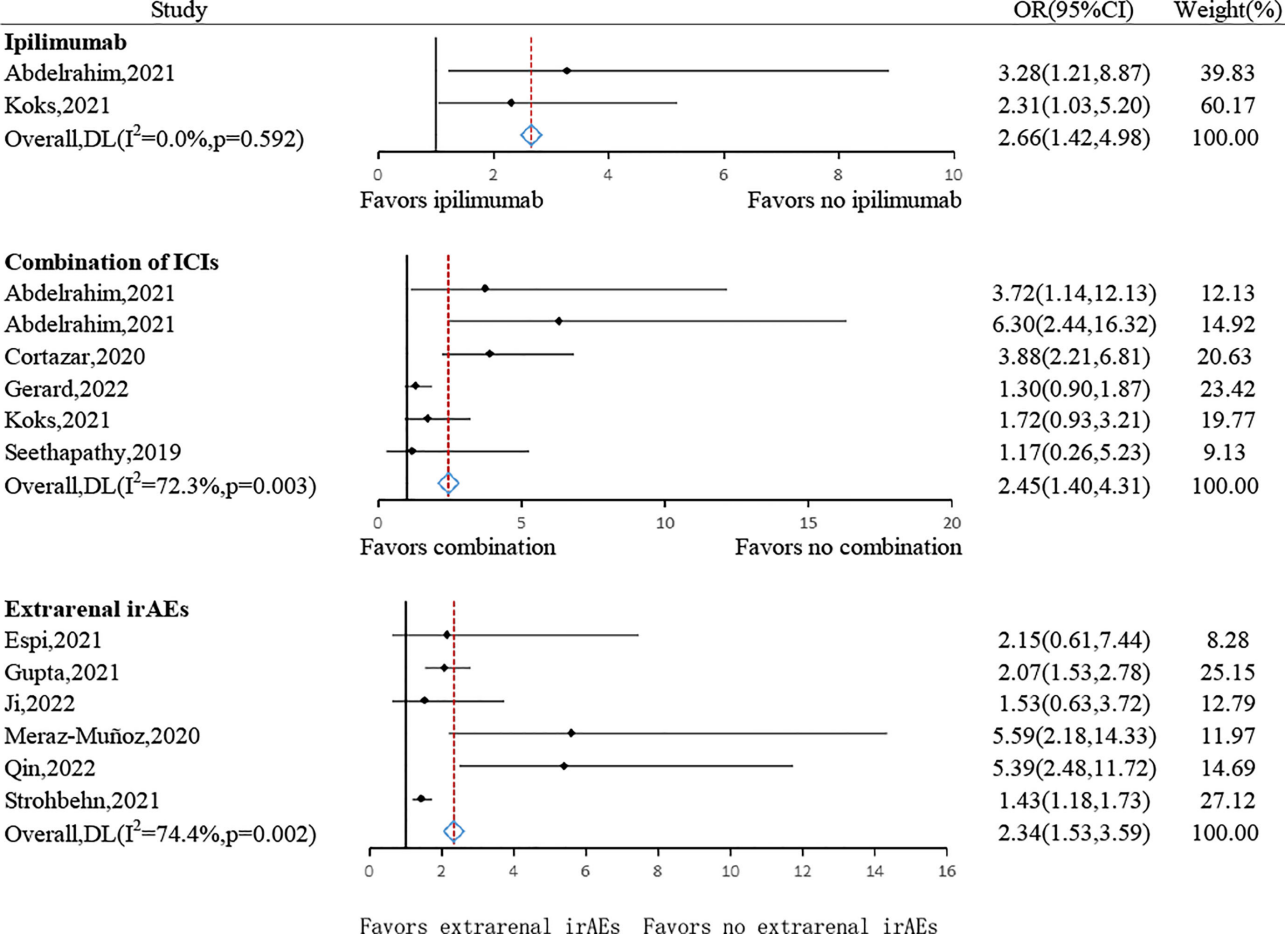
Forest plots of the odds ratio for type of ICIs and extrarenal irAEs.

The risk of AKI following ICIs was significantly higher in cancerous individuals receiving ipilimumab (pooled OR: 2.66, 95% CI: 1.42–4.98, *P*= 0.002, *I* ^2^ = 0.0%, n= 2 studies) or who received combined treatment with two or more kinds of ICIs (pooled OR: 2.45, 95% CI: 1.40–4.31, *P*= 0.002, *I* ^2^ = 72.3%, n= 6 studies) (**Figure 5**). The risk of patients receiving pembrolizumab was not statistically different (pooled OR: 2.17, 95% CI: 0.90–5.21, *P*= 0.083, *I* ^2^ = 60.0%, n= 2 studies) (**Supplementary Figure S7**). In addition, no substantial difference between the duration or cycle of ICI treatment and the risk of ICI-AKI was found (pooled OR: 1.17, 95% CI: 0.92-1.47, *P*= 0.198, *I* ^2^ = 83.2%, n= 3 studies) (**Supplementary Figure S6**).

The presence of extrarenal irAEs such as colitis, hypophysitis, rash, and pneumonitis was strongly suggested as an important risk factor for AKI (pooled OR: 2.34, 95% CI: 1.53–3.59, *P*<0.001, *I* ^2^ = 74.4%, n= 6 studies) (**Figure 5**) (33).

#### 3.4.3 Concomitant agents

**Figure 6:**
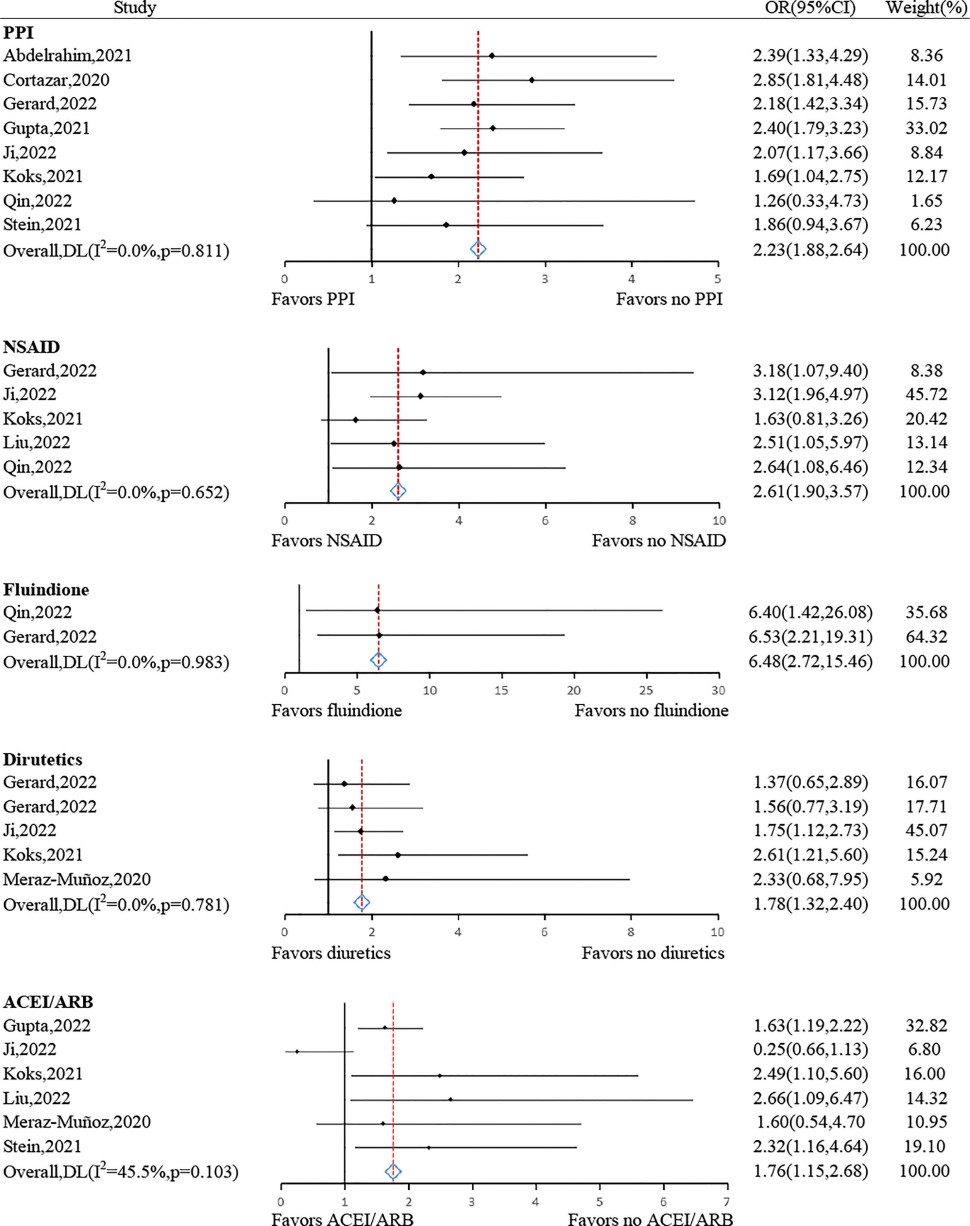
Forest plots of the odds ratio for concomitant agents.

Various concomitant agents administered accompanied by ICIs were associated with a significantly greater risk of developing AKI, including PPIs (pooled OR: 2.23, 95% CI: 1.88–2.64, *P*< 0.001, *I* ^2^ = 0.0%, n= 8 studies), NSAID (pooled OR: 2.61, 95% CI: 1.90–3.57, *P*< 0.001, *I* ^2^ = 0.0%, n = 5 studies), fluindione (pooled OR: 6.48, 95% CI: 2.72–15.46, *P*< 0.001, *I* ^2^ = 0.0%, n = 2 studies), diuretics (pooled OR: 1.78, 95% CI: 1.32–2.40, *P*< 0.001, *I* ^2^ = 0.0%, n= 5 studies) and ACEI/ARB (pooled OR: 1.76, 95% CI: 1.15–2.68, *P*= 0.009, *I* ^2^ = 45.5%, n= 6 studies) (**Figure 6**).

#### 3.4.4 Sensitivity analysis and publication bias

**Figure 7:**
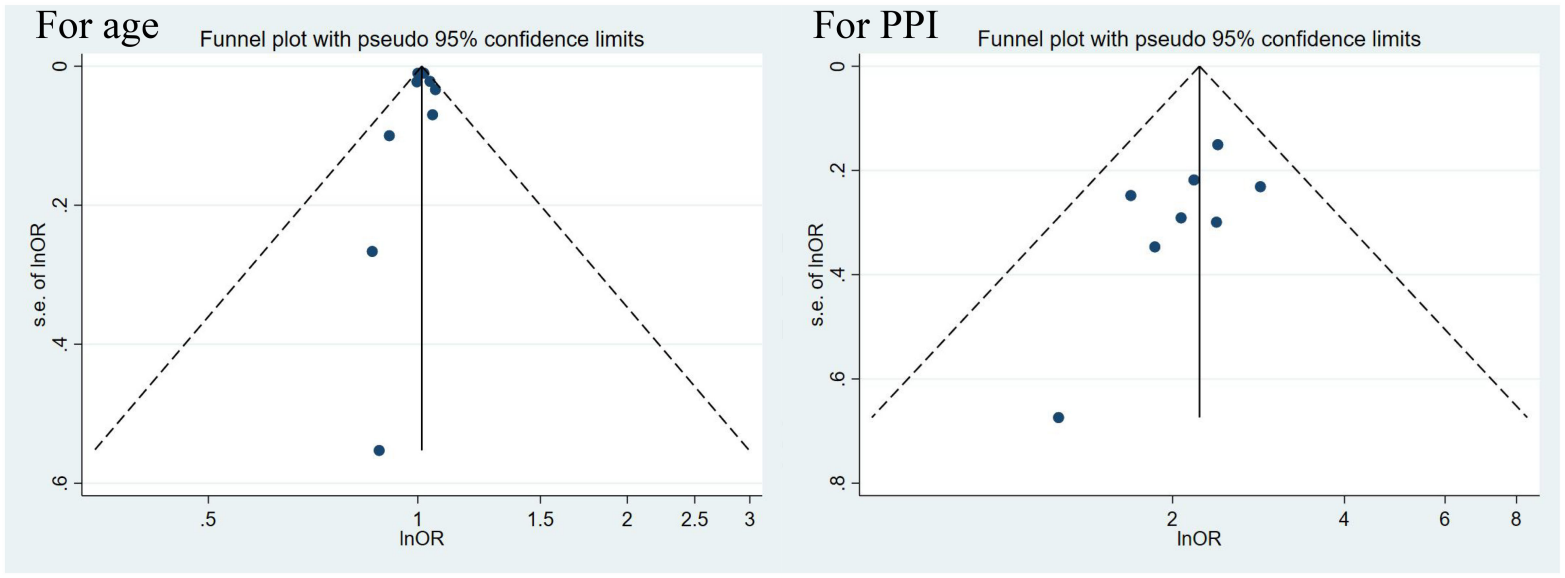
Funnel plots of age and PPI.

In consideration of adequate studies on age and PPI as risk factors, sensitivity and publication bias analyses were performed to verify the stability of their results. We conducted sensitivity analyses by omitting 1 study at a time and recalculating the estimates on the remaining studies. No single study was observed to influence the overall estimates (**Supplementary Table S2**). According to the publication bias analyses, graphical symmetry presented by the funnel plot was satisfied, and *P* was more than 0.05 for both Begg’s test and Egger’s test, confirming robust results (**Figure 7** and **Supplementary Table 3**).

### 3.5 Time from ICIs initiation to ICI-AKI

Among the selected studies, there were 4 studies investigating median time from first ICI administration to the occurrence of ICI-AKI, ranging from 88.9 days to 112 days. The overall pooled median time was 108.07 days (**Supplementary Figure S8**).

## 4 Discussion

### 4.1 Principal findings and clinical interpretation

This systematic review and meta-analysis is one of the first to comprehensively reveal the incidence and substantial risk factors of AKI secondary to ICIs in patients suffering from cancer. The overall estimated pooled incidence was 5.7% based on 22 included studies. The therapeutic effects of ICIs for malignances are often accompanied by unavoidable damage to innocent organs such as the kidney due to imprecise strikes in the body (38, 39). In contrast to the previous recognition that AKI secondary to ICIs was relatively rare, with a morbidity of 1.3% in a meta-analysis conducted in 2022 (40), the overall pooled incidence of AKI was as high as 5.7% in our meta-analysis, demonstrating a greatly underrated result in the past. This inconsistency could be due to the expanding use of ICI in the latest years among vulnerable patient group, the growing awareness of ICI-related adverse effects, as well as the ambiguous definition of ICI-AKI. Only severe and fatal AKI was detected when it was reported initially, leading to an underestimation of the overall incidence at early ages. In addition, the occurrences of AKI were all attributed to ICIs in some of the included studies regardless of any other potential reason, largely responsible for uncertainties in the incidence of ICI-AKI. Given these considerations, a unified conception and early diagnosis of ICI-AKI in cancer patients are imperative for accurate estimation of the incidence. Effort to address the scarcity of representative estimates has been made by Gupta et al., who introduced a relatively clear definition of ICI-AKI based on at least one of the following: acute tubulointerstitial nephritis on kidney biopsy, received ICI therapy for at least one cycle, but this definition has not been applied widely (20). Respecting the noted incidence and high mortality rate of 15.9% for AKI after ICIs (8), proper diagnosis and refined management of this anticancer treatment-induced AKI is a challenge for both oncologists and nephrologists due to their limited clinical skills and knowledge. However, compared with other therapies against tumors with renal-associated adverse outcomes, such as small-molecule protein kinase inhibitors (PKIs), whose incidence of secondary AKI is 2.16% and corresponding mortality is 26% (41), ICIs are advantageous in terms of ultimate survival outcomes. Hence, risk factors for secondary AKI due to ICIs need to be comprehensively analyzed and applied to make individualized decisions for patients with potential risk factors and predict the development of AKI. In this random-effect meta-analysis, we extracted 15 potential risk factors investigated by at least 2 studies to be statistically analyzed. Patients with older age and preexisting CKD were at increased risk of AKI during ICI treatment. In addition, ipilimumab, the combined use of 2 or more ICIs and extrarenal irAEs were also contributors to ICI-AKI. Seethapathy et al. found that patients receiving CTLA-4, such as ipilimumab, showed a higher risk of developing AKI than those receiving PD-1 like pembrolizumab, and patients treated with PD-L1 had a similar tendency to PD-1 but have not been fully proven statistically, which was not included in this meta-analysis because no other studies have emphasized it (11). The most frequent organs affected by ICIs are skin, liver, heart, lungs and so on (42), whose impairments are significant indicators of the occurrence of ICI-AKI as extrarenal irAEs are associated with ICI-AKI. Concomitant agents are of great importance to the occurrence of AKI, among which PPI, NSAID, fluindione, diuretics and ACEI/ARB use were proven to be common risk factors. Therefore, clinicians are supposed to be cautious and evaluate the pros and cons when treating cancer patients who are receiving those therapies with ICIs. In addition to the key risk factors listed hereinbefore, other potential risk factors not included in this meta-analysis due to limited data available are displayed in **Supplementary Table S4**. The median time of the occurrence AKI after ICIs initiation was found to be 108.07 days by our study, in contrast to a 2022 pharmacovigilance study reporting a time interval of 48 days which however, did not clearly point out whether it was calculated from the first dose or last dose of ICIs (8).

With regard to the underlying pathogenesis of ICI-AKI, we have learned that ICIs such as anti-PD-1 and anti-CTLA-4 could activate the immune system by detecting and combining with immune checkpoints on the surface of immune cells such as T cells, B cells, and dendritic cells to prevent inhibited activation by B7 and PD-L1/PD-L2, which function as regulators of immune tolerance. AKI induced by ICIs stands a good chance of being deprived of this protective mechanism in the kidney caused by reprogramming of the immune system. Abnormally activated T cells have a tendency to attack renal cells when provoked by exogenous or endogenous factors, which also explains the result of this meta-analysis that patients with concomitant PPIs, NSAIDs and other drugs that could induce acute interstitial nephritis (AIN) are at greater risk of AKI (43). PPIs and NSAIDs can bind to renal tubules and stimulate immune responses that are suppressed under normal conditions. Interestingly, even kidney tissue can be recognized as a natural antigen (44). Therefore, renal cells are attacked by T cells activated by ICIs and the stimulation of the immune system by concomitant agents or the kidney, resulting in AKI (45). AIN was the most common lesion on kidney biopsy of ICI-AKI, demonstrating that inflammation may be an important contributor to the development of AKI in cancer patients treated with ICIs (19). Elevated levels of serum pro-inflammatory cytokines/chemokines, such as IL-1Ra and TNF-α, were detected during pharmacological treatment with ICIs (46), which may result in AKI when they immigrate to renal tissue. Extrarenal irAEs as risk factors for AKI might be explained by the multisystemic responses brought about by inflammatory factors, although this hypothesis has not yet been confirmed. The potential reason for the higher risk of AKI observed in patients receiving ipilimumab compared with other ICI drugs of anti-PD-L1 is likely due to its identity as an anti-CTLA-4 agent. In contrast, PD-L1 is expressed on renal tubular epithelial cells and may play an inhibitory role in immunopathogenesis in the kidney, although its understanding remains poor (47).

### 4.2 Strengths and limitations

This systematic review and meta-analysis filled the gap of insufficient and not comprehensive data on AKI following ICIs with regard to incidence and risk factors, as well as latency period of ICI-AKI. Several limitations should be noted when interpreting the results of this study. First, publications enrolled in this meta-analysis were mostly retrospective cohort studies. Potential bias is unavoidable due to the nature of source studies. Second, the definition of ICI-AKI varied across studies. A universal diagnostic criterium of ICI-AKI is needed. Third, the number of studies included in the analysis of certain risk factors was limited, which might hamper the robustness of the study findings, especially for preexisting CKD, ipilimumab and fluindione investigated by only 2 studies. In addition, as only a small fraction of the primary studies reported tumor-type specific data of AKI following ICI use, we were unable to perform subgroup analysis stratified by type of tumor.

### 4.3 Future directions

Some potential risk factors were not included in this meta-analysis due to the limited number of primary studies. For example, Asian participants were found by Abdelrahim et al. to have a stronger tendency to contract AKI secondary to ICIs (12). In terms of comorbidities, anemia, Alb<30 g/L or liver disease, gynecologic cancer and ICI-induced thyroiditis were reported as risk factors by only one study (19) (20) (46). More future studies are warranted to verify these candidate risk factors. Apart from them, the influence of tumor type on the occurrence of ICI-AKI also deserves further investigation. More importantly, a clear and universal definition of ICI-AKI would be helpful to standardize future research and optimize patient management.

## 5 Conclusions

This systematic review and meta-analysis demonstrated a substantial incidence (5.7%) of AKI in cancer patients receiving ICIs with a median latency period of 108.07 days. Older age, preexisting CKD, ipilimumab, joint use of more than one ICI, extrarenal irAEs, and use of PPI, NSAID, fluindione, diuretics and ACEI/ARB were all identified as risk factors for ICI-AKI. These findings may provide insights into the optimization of the management of patients who receive ICI treatment.

## Data availability statement

The original contributions presented in the study are included in the article/**Supplementary Material**. Further inquiries can be directed to the corresponding author.

## Author contributions

Conceptualization: YZ, LZ and PF. Data Curation: YZ, CL, WW, LY, and CY. Formal analysis: YZ, CL, WW, YP, TY, and FN. Funding Acquisition: YZ. Supervision: YZ and PF. Writing-original draft: CL and YZ. Writing review/editing: all co-authors.
