# Supplementary material for: Incidence and risk factors of acute kidney injury in cancer patients treated with immune checkpoint inhibitors: a systematic review and meta-analysis
Source: Front Immunol. 2023 May 29;14:1173952. doi: 10.3389/fimmu.2023.1173952 (PMC10258324; doi:10.3389/fimmu.2023.1173952)
Supplement: Supplementary file 1 [file DataSheet_1.docx]

**Appendix S1**

**Search strategy for incidence:**

**PubMed/Medline**

(((((((((((((((((((((((((((((((((((("Immune Checkpoint Inhibitors"[Mesh]) ) OR (Checkpoint Inhibitors, Immune[Title/Abstract])) OR (Immune Checkpoint Inhibitor[Title/Abstract])) OR (Checkpoint Inhibitor, Immune[Title/Abstract])) OR (Immune Checkpoint Blockers[Title/Abstract])) OR (Checkpoint Blockers, Immune[Title/Abstract])) OR (Immune Checkpoint Blockade[Title/Abstract])) OR (Checkpoint Blockade, Immune[Title/Abstract])) OR (Immune Checkpoint Inhibition[Title/Abstract])) OR (Checkpoint Inhibition, Immune[Title/Abstract])) OR (PD-L1 Inhibitors[Title/Abstract])) OR (PD L1 Inhibitors[Title/Abstract])) OR (PD-L1 Inhibitor[Title/Abstract])) OR (PD L1 Inhibitor[Title/Abstract])) OR (Programmed Death-Ligand 1 Inhibitors[Title/Abstract])) OR (Programmed Death Ligand 1 Inhibitors[Title/Abstract])) OR (PD-1-PD-L1 Blockade[Title/Abstract])) OR (Blockade, PD-1-PD-L1[Title/Abstract])) OR (PD 1 PD L1 Blockade[Title/Abstract])) OR (CTLA-4 Inhibitors[Title/Abstract])) OR (CTLA 4 Inhibitors[Title/Abstract])) OR (CTLA-4 Inhibitor[Title/Abstract])) OR (CTLA 4 Inhibitor[Title/Abstract])) OR (Cytotoxic T-Lymphocyte-Associated Protein 4 Inhibitors[Title/Abstract])) OR (Cytotoxic T Lymphocyte Associated Protein 4 Inhibitors[Title/Abstract])) OR (Cytotoxic T-Lymphocyte-Associated Protein 4 Inhibitor[Title/Abstract])) OR (Cytotoxic T Lymphocyte Associated Protein 4 Inhibitor[Title/Abstract])) OR (PD-1 Inhibitors[Title/Abstract])) OR (PD 1 Inhibitors[Title/Abstract])) OR (PD-1 Inhibitor[Title/Abstract])) OR (Inhibitor, PD-1[Title/Abstract])) OR (PD 1 Inhibitor[Title/Abstract])) OR (Programmed Cell Death Protein 1 Inhibitor[Title/Abstract])) OR (Programmed Cell Death Protein 1 Inhibitors[Title/Abstract])) AND (("Incidence"[Mesh]) OR (((((((((((((((((((Incidences[Title/Abstract]) OR (Secondary Attack Rate[Title/Abstract])) OR (Attack Rate, Secondary[Title/Abstract])) OR (Rate, Secondary Attack[Title/Abstract])) OR (Secondary Attack Rates[Title/Abstract])) OR (Incidence Proportion[Title/Abstract])) OR (Incidence Proportions[Title/Abstract])) OR (Proportion, Incidence[Title/Abstract])) OR (Attack Rate[Title/Abstract])) OR (Attack Rates[Title/Abstract])) OR (Rate, Attack[Title/Abstract])) OR (Cumulative Incidence[Title/Abstract])) OR (Cumulative Incidences[Title/Abstract])) OR (Incidence, Cumulative[Title/Abstract])) OR (Incidence Rates[Title/Abstract])) OR (Rate, Incidence[Title/Abstract])) OR (Person-time Rate[Title/Abstract])) OR (Person-time Rates[Title/Abstract])) OR (Rate, Person-time[Title/Abstract])))) AND (((((((((((((((((((((((("Acute Kidney Injury"[Mesh]) OR (Acute Kidney Injuries[Title/Abstract])) OR (Kidney Injuries, Acute[Title/Abstract])) OR (Kidney Injury, Acute[Title/Abstract])) OR (Acute Renal Injury[Title/Abstract])) OR (Acute Renal Injuries[Title/Abstract])) OR (Renal Injuries, Acute[Title/Abstract])) OR (Renal Injury, Acute[Title/Abstract])) OR (Kidney Failure, Acute[Title/Abstract])) OR (Acute Kidney Failures[Title/Abstract])) OR (Kidney Failures, Acute[Title/Abstract])) OR (Acute Renal Failure[Title/Abstract])) OR (Acute Renal Failures[Title/Abstract])) OR (Renal Failures, Acute[Title/Abstract])) OR (Renal Failure, Acute[Title/Abstract])) OR (Acute Kidney Failure[Title/Abstract])) OR (Renal Insufficiency, Acute[Title/Abstract])) OR (Acute Renal Insufficiencies[Title/Abstract])) OR (Renal Insufficiencies, Acute[Title/Abstract])) OR (Acute Renal Insufficiency[Title/Abstract])) OR (Kidney Insufficiency, Acute[Title/Abstract])) OR (Acute Kidney Insufficiencies[Title/Abstract])) OR (Kidney Insufficiencies, Acute[Title/Abstract])) OR (Acute Kidney Insufficiency[Title/Abstract]))

**Cochrane**

ID Search Hits

#1 MeSH descriptor: [Incidence] explode all trees 12572

#2 ((Incidences or Secondary Attack Rate or Attack Rate, Secondary or Rate, Secondary Attack or Secondary Attack Rates or Incidence Proportion or Incidence Proportions or Proportion, Incidence or Attack Rate or Attack Rates or Rate, Attack or Cumulative Incidence or Cumulative Incidences or Incidence, Cumulative or Incidence Rates or Rate, Incidence or Person-time Rate or Person-time Rates or Rate, Person-time)):ti,ab,kw (Word variations have been searched) 150091

#3 #1 or #2 150091

#4 MeSH descriptor: [Acute Kidney Injury] explode all trees 1966

#5 (Acute Kidney Injuries or Kidney Injuries, Acute or Kidney Injury, Acute or Acute Renal Injury or Acute Renal Injuries or Renal Injuries, Acute or Renal Injury, Acute or Kidney Failure, Acute or Acute Kidney Failures or Kidney Failures, Acute or Acute Renal Failure or Acute Renal Failures or Renal Failures, Acute or Renal Failure, Acute or Acute Kidney Failure or Renal Insufficiency, Acute or Acute Renal Insufficiencies or Renal Insufficiencies, Acute or Acute Renal Insufficiency or Kidney Insufficiency, Acute or Acute Kidney Insufficiencies or Kidney Insufficiencies, Acute or Acute Kidney Insufficiency):ti,ab,kw (Word variations have been searched) 11103

#6 #4 or #5 11127

#7 MeSH descriptor: [Immune Checkpoint Inhibitors] explode all trees 177

#8 (Checkpoint Inhibitors, Immune or Immune Checkpoint Inhibitor or Checkpoint Inhibitor, Immune or Immune Checkpoint Blockers or Checkpoint Blockers, Immune or Immune Checkpoint Blockade or Checkpoint Blockade, Immune or Immune Checkpoint Inhibition or Checkpoint Inhibition, Immune or PD L1 Inhibitors or PD L1 Inhibitors or PD L1 Inhibitor or PD L1 Inhibitor or Programmed Death Ligand 1 Inhibitors or Programmed Death Ligand 1 Inhibitors or PD 1 PD L1 Blockade or Blockade, PD 1 PD L1 or PD 1 PD L1 Blockade or CTLA 4 Inhibitors or CTLA 4 Inhibitors or CTLA 4 Inhibitor or CTLA 4 Inhibitor or Cytotoxic T Lymphocyte Associated Protein 4 Inhibitors or Cytotoxic T Lymphocyte Associated Protein 4 Inhibitors or Cytotoxic T Lymphocyte Associated Protein 4 Inhibitor or Cytotoxic T Lymphocyte Associated Protein 4 Inhibitor or PD 1 Inhibitors or PD 1 Inhibitors or PD 1 Inhibitor or Inhibitor, PD 1 or PD 1 Inhibitor or Programmed Cell Death Protein 1 Inhibitor or Programmed Cell Death Protein 1 Inhibitors):ti,ab,kw (Word variations have been searched) 7335

#9 #7 or #8 7335

#10 #3 and #6 3074

#11 #10 and #9 25

**Web of science**

(TS=(Acute Kidney Injury) OR AB=(AKI OR Acute Kidney Injuries OR Kidney Injuries, Acute OR Kidney Injury, Acute OR Acute Renal Injury OR Acute Renal Injuries OR Renal Injuries, Acute OR Renal Injury, Acute OR Kidney Failure, Acute OR Acute Kidney Failures OR Kidney Failures, Acute OR Acute Renal Failure OR Acute Renal Failures OR Renal Failures, Acute OR Renal Failure, Acute OR Acute Kidney Failure OR Renal Insufficiency, Acute OR Acute Renal Insufficiencies OR Renal Insufficiencies, Acute OR Acute Renal Insufficiency OR Kidney Insufficiency, Acute OR Acute Kidney Insufficiencies OR Kidney Insufficiencies, Acute OR Acute Kidney Insufficiency)) AND (TS=(Immune Checkpoint Inhibitors ) OR AB=(Checkpoint Inhibitors, Immune OR Immune Checkpoint Inhibitor OR Checkpoint Inhibitor, Immune OR Immune Checkpoint Blockers OR Checkpoint Blockers, Immune OR Immune Checkpoint Blockade OR Checkpoint Blockade, Immune OR Immune Checkpoint Inhibition OR Checkpoint Inhibition, Immune OR PD-L1 Inhibitors OR PD L1 Inhibitors OR PD-L1 Inhibitor OR PD L1 Inhibitor OR Programmed Death-Ligand 1 Inhibitors OR Programmed Death Ligand 1 Inhibitors OR PD-1-PD-L1 Blockade OR Blockade, PD-1-PD-L1 OR PD 1 PD L1 Blockade OR CTLA-4 Inhibitors OR CTLA 4 Inhibitors OR CTLA-4 Inhibitor OR CTLA 4 Inhibitor OR Cytotoxic T-Lymphocyte-Associated Protein 4 Inhibitors OR Cytotoxic T Lymphocyte Associated Protein 4 Inhibitors OR Cytotoxic T-Lymphocyte-Associated Protein 4 Inhibitor OR Cytotoxic T Lymphocyte Associated Protein 4 Inhibitor OR PD-1 Inhibitors OR PD 1 Inhibitors OR PD-1 Inhibitor OR Inhibitor, PD-1 OR PD 1 Inhibitor OR Programmed Cell Death Protein 1 Inhibitor OR Programmed Cell Death Protein 1 Inhibitors)) AND (TS=(Incidence) OR AB=(Incidences OR Secondary Attack Rate OR Attack Rate, Secondary OR Rate, Secondary Attack OR Secondary Attack Rates OR Incidence Proportion OR Incidence Proportions OR Proportion, Incidence OR Attack Rate OR Attack Rates OR Rate, Attack OR Cumulative Incidence OR Cumulative Incidences OR Incidence, Cumulative OR Incidence Rates OR Rate, Incidence OR Person-time Rate OR Person-time Rates OR Rate, Person-time))

**Embase**

#10. #3 AND #6 AND #9 176

#9. #7 OR #8 979,866 14 Feb 2023

#8. 'incidence'/exp 640,839

#7. incidences OR 'secondary attack rate'/exp OR 979,866 'secondary attack rate' OR (('secondary'/exp OR

secondary) AND attack AND rate) OR 'attack rate,

secondary' OR (attack AND rate, AND

('secondary'/exp OR secondary)) OR 'rate,

secondary attack' OR (rate, AND ('secondary'/exp

OR secondary) AND attack) OR 'secondary attack

rates' OR (('secondary'/exp OR secondary) AND

attack AND rates) OR 'incidence proportion'/exp

OR 'incidence proportion' OR (('incidence'/exp OR

incidence) AND proportion) OR 'incidence

proportions' OR (('incidence'/exp OR incidence)

AND proportions) OR 'proportion, incidence' OR

(proportion, AND ('incidence'/exp OR incidence))

OR 'attack rate'/exp OR 'attack rate' OR (attack

AND rate) OR 'attack rates' OR (attack AND rates)

OR 'rate, attack' OR (rate, AND attack) OR

'cumulative incidence'/exp OR 'cumulative

incidence' OR (cumulative AND ('incidence'/exp OR

incidence)) OR 'cumulative incidences' OR

(cumulative AND incidences) OR 'incidence,

cumulative' OR (('incidence,'/exp OR incidence,)

AND cumulative) OR 'incidence rates' OR

(('incidence'/exp OR incidence) AND rates) OR

'rate, incidence'/exp OR 'rate, incidence' OR

(rate, AND ('incidence'/exp OR incidence)) OR

'person-time rate'/exp OR 'person-time rate' OR

('person time' AND rate) OR 'person-time rates'

OR ('person time' AND rates) OR 'rate,

person-time' OR (rate, AND 'person time')

#6. #4 OR #5 113,404

#5. 'immune checkpoint inhibitor'/exp 19,147

#4. 'checkpoint inhibitors, immune' OR (checkpoint 113,404 AND inhibitors, AND ('immune'/exp OR immune)) OR

'immune checkpoint inhibitor'/exp OR 'immune

checkpoint inhibitor' OR (('immune'/exp OR

immune) AND checkpoint AND ('inhibitor'/exp OR

inhibitor)) OR 'checkpoint inhibitor, immune' OR

(checkpoint AND inhibitor, AND ('immune'/exp OR

immune)) OR 'immune checkpoint blockers' OR

(('immune'/exp OR immune) AND checkpoint AND

blockers) OR 'checkpoint blockers, immune' OR

(checkpoint AND blockers, AND ('immune'/exp OR

immune)) OR 'immune checkpoint blockade'/exp OR

'immune checkpoint blockade' OR (('immune'/exp OR

immune) AND checkpoint AND blockade) OR

'checkpoint blockade, immune' OR (checkpoint AND

blockade, AND ('immune'/exp OR immune)) OR

'immune checkpoint inhibition'/exp OR 'immune

checkpoint inhibition' OR (('immune'/exp OR

immune) AND checkpoint AND ('inhibition'/exp OR

inhibition)) OR 'checkpoint inhibition, immune'

OR (checkpoint AND inhibition, AND ('immune'/exp

OR immune)) OR 'pd-l1 inhibitors' OR ('pd l1' AND

('inhibitors'/exp OR inhibitors)) OR 'pd l1

inhibitors' OR (('pd'/exp OR pd) AND ('l1'/exp OR

l1) AND ('inhibitors'/exp OR inhibitors)) OR

'pd-l1 inhibitor' OR ('pd l1' AND

('inhibitor'/exp OR inhibitor)) OR 'pd l1

inhibitor'/exp OR 'pd l1 inhibitor' OR (('pd'/exp

OR pd) AND ('l1'/exp OR l1) AND ('inhibitor'/exp

OR inhibitor)) OR 'programmed death-ligand 1

inhibitors' OR (programmed AND 'death ligand' AND

('1'/exp OR 1) AND ('inhibitors'/exp OR

inhibitors)) OR 'programmed death ligand 1

inhibitors' OR (programmed AND ('death'/exp OR

death) AND ('ligand'/exp OR ligand) AND ('1'/exp

OR 1) AND ('inhibitors'/exp OR inhibitors)) OR

'pd-1-pd-l1 blockade' OR ('pd 1 pd l1' AND

blockade) OR 'blockade, pd-1-pd-l1' OR (blockade,

AND 'pd 1 pd l1') OR 'pd 1 pd l1 blockade' OR

(('1'/exp OR 1) AND ('pd'/exp OR pd) AND

('l1'/exp OR l1) AND blockade) OR 'ctla-4

inhibitors' OR (('ctla 4'/exp OR 'ctla 4') AND

('inhibitors'/exp OR inhibitors)) OR 'ctla 4

inhibitors' OR (ctla AND 4 AND ('inhibitors'/exp

OR inhibitors)) OR 'ctla-4 inhibitor' OR (('ctla

4'/exp OR 'ctla 4') AND ('inhibitor'/exp OR

inhibitor)) OR 'ctla 4 inhibitor' OR (ctla AND 4

AND ('inhibitor'/exp OR inhibitor)) OR 'cytotoxic

t-lymphocyte-associated protein 4 inhibitors' OR

(cytotoxic AND 't lymphocyte associated' AND

('protein'/exp OR protein) AND 4 AND

('inhibitors'/exp OR inhibitors)) OR 'cytotoxic t

lymphocyte associated protein 4 inhibitors' OR

(cytotoxic AND t AND ('lymphocyte'/exp OR

lymphocyte) AND associated AND ('protein'/exp OR

protein) AND 4 AND ('inhibitors'/exp OR

inhibitors)) OR 'cytotoxic

t-lymphocyte-associated protein 4 inhibitor' OR

(cytotoxic AND 't lymphocyte associated' AND

('protein'/exp OR protein) AND 4 AND

('inhibitor'/exp OR inhibitor)) OR 'cytotoxic t

lymphocyte associated protein 4 inhibitor' OR

(cytotoxic AND t AND ('lymphocyte'/exp OR

lymphocyte) AND associated AND ('protein'/exp OR

protein) AND 4 AND ('inhibitor'/exp OR

inhibitor)) OR 'pd-1 inhibitors' OR (('pd 1'/exp

OR 'pd 1') AND ('inhibitors'/exp OR inhibitors))

OR 'pd 1 inhibitors' OR (('pd'/exp OR pd) AND

('1'/exp OR 1) AND ('inhibitors'/exp OR

inhibitors)) OR 'pd-1 inhibitor' OR (('pd 1'/exp

OR 'pd 1') AND ('inhibitor'/exp OR inhibitor)) OR

'inhibitor, pd-1' OR (inhibitor, AND ('pd 1'/exp

OR 'pd 1')) OR 'pd 1 inhibitor'/exp OR 'pd 1

inhibitor' OR (('pd'/exp OR pd) AND ('1'/exp OR

1) AND ('inhibitor'/exp OR inhibitor)) OR

'programmed cell death protein 1 inhibitor'/exp

OR 'programmed cell death protein 1 inhibitor' OR

(programmed AND ('cell'/exp OR cell) AND

('death'/exp OR death) AND ('protein'/exp OR

protein) AND ('1'/exp OR 1) AND ('inhibitor'/exp

OR inhibitor)) OR 'programmed cell death protein

1 inhibitors' OR (programmed AND ('cell'/exp OR

cell) AND ('death'/exp OR death) AND

('protein'/exp OR protein) AND ('1'/exp OR 1) AND

('inhibitors'/exp OR inhibitors))

#3. #1 OR #2 278,728

#2. 'acute kidney failure'/exp 120,531

#1. aki OR 'acute kidney injuries' OR (acute AND 278,728

('kidney'/exp OR kidney) AND ('injuries'/exp OR

injuries)) OR 'kidney injuries, acute' OR

(('kidney'/exp OR kidney) AND ('injuries,'/exp OR

injuries,) AND acute) OR 'kidney injury, acute'

OR (('kidney'/exp OR kidney) AND ('injury,'/exp

OR injury,) AND acute) OR 'acute renal

injury'/exp OR 'acute renal injury' OR (acute AND

('renal'/exp OR renal) AND ('injury'/exp OR

injury)) OR 'acute renal injuries' OR (acute AND

('renal'/exp OR renal) AND ('injuries'/exp OR

injuries)) OR 'renal injuries, acute' OR

(('renal'/exp OR renal) AND ('injuries,'/exp OR

injuries,) AND acute) OR 'renal injury, acute' OR

(('renal'/exp OR renal) AND ('injury,'/exp OR

injury,) AND acute) OR 'kidney failure,

acute'/exp OR 'kidney failure, acute' OR

(('kidney'/exp OR kidney) AND failure, AND acute)

OR 'acute kidney failures' OR (acute AND

('kidney'/exp OR kidney) AND failures) OR 'kidney

failures, acute' OR (('kidney'/exp OR kidney) AND

failures, AND acute) OR 'acute renal failure'/exp

OR 'acute renal failure' OR (acute AND

('renal'/exp OR renal) AND ('failure'/exp OR

failure)) OR 'acute renal failures' OR (acute AND

('renal'/exp OR renal) AND failures) OR 'renal

failures, acute' OR (('renal'/exp OR renal) AND

failures, AND acute) OR 'renal failure, acute' OR

(('renal'/exp OR renal) AND failure, AND acute)

OR 'acute kidney failure'/exp OR 'acute kidney

failure' OR (acute AND ('kidney'/exp OR kidney)

AND ('failure'/exp OR failure)) OR 'renal

insufficiency, acute'/exp OR 'renal

insufficiency, acute' OR (('renal'/exp OR renal)

AND insufficiency, AND acute) OR 'acute renal

insufficiencies' OR (acute AND ('renal'/exp OR

renal) AND insufficiencies) OR 'renal

insufficiencies, acute' OR (('renal'/exp OR

renal) AND insufficiencies, AND acute) OR 'acute

renal insufficiency'/exp OR 'acute renal

insufficiency' OR (acute AND ('renal'/exp OR

renal) AND insufficiency) OR 'kidney

insufficiency, acute'/exp OR 'kidney

insufficiency, acute' OR (('kidney'/exp OR

kidney) AND insufficiency, AND acute) OR 'acute

kidney insufficiencies' OR (acute AND

('kidney'/exp OR kidney) AND insufficiencies) OR

'kidney insufficiencies, acute' OR (('kidney'/exp

OR kidney) AND insufficiencies, AND acute) OR

'acute kidney insufficiency'/exp OR 'acute kidney

insufficiency' OR (acute AND ('kidney'/exp OR

kidney) AND insufficiency)

**Appendix S2**

**Search strategy for risk factors:**

**PubMed/Medline**

(((((((((((((((((((((((((((((((((((("Immune Checkpoint Inhibitors"[Mesh]) ) OR (Checkpoint Inhibitors, Immune[Title/Abstract])) OR (Immune Checkpoint Inhibitor[Title/Abstract])) OR (Checkpoint Inhibitor, Immune[Title/Abstract])) OR (Immune Checkpoint Blockers[Title/Abstract])) OR (Checkpoint Blockers, Immune[Title/Abstract])) OR (Immune Checkpoint Blockade[Title/Abstract])) OR (Checkpoint Blockade, Immune[Title/Abstract])) OR (Immune Checkpoint Inhibition[Title/Abstract])) OR (Checkpoint Inhibition, Immune[Title/Abstract])) OR (PD-L1 Inhibitors[Title/Abstract])) OR (PD L1 Inhibitors[Title/Abstract])) OR (PD-L1 Inhibitor[Title/Abstract])) OR (PD L1 Inhibitor[Title/Abstract])) OR (Programmed Death-Ligand 1 Inhibitors[Title/Abstract])) OR (Programmed Death Ligand 1 Inhibitors[Title/Abstract])) OR (PD-1-PD-L1 Blockade[Title/Abstract])) OR (Blockade, PD-1-PD-L1[Title/Abstract])) OR (PD 1 PD L1 Blockade[Title/Abstract])) OR (CTLA-4 Inhibitors[Title/Abstract])) OR (CTLA 4 Inhibitors[Title/Abstract])) OR (CTLA-4 Inhibitor[Title/Abstract])) OR (CTLA 4 Inhibitor[Title/Abstract])) OR (Cytotoxic T-Lymphocyte-Associated Protein 4 Inhibitors[Title/Abstract])) OR (Cytotoxic T Lymphocyte Associated Protein 4 Inhibitors[Title/Abstract])) OR (Cytotoxic T-Lymphocyte-Associated Protein 4 Inhibitor[Title/Abstract])) OR (Cytotoxic T Lymphocyte Associated Protein 4 Inhibitor[Title/Abstract])) OR (PD-1 Inhibitors[Title/Abstract])) OR (PD 1 Inhibitors[Title/Abstract])) OR (PD-1 Inhibitor[Title/Abstract])) OR (Inhibitor, PD-1[Title/Abstract])) OR (PD 1 Inhibitor[Title/Abstract])) OR (Programmed Cell Death Protein 1 Inhibitor[Title/Abstract])) OR (Programmed Cell Death Protein 1 Inhibitors[Title/Abstract])) AND ((((((((((((((((((("Risk Factors"[Mesh]) OR (Factor, Risk[Title/Abstract])) OR (Risk Factor[Title/Abstract])) OR (Social Risk Factors[Title/Abstract])) OR (Factor, Social Risk[Title/Abstract])) OR (Factors, Social Risk[Title/Abstract])) OR (Risk Factor, Social[Title/Abstract])) OR (Risk Factors, Social[Title/Abstract])) OR (Social Risk Factor[Title/Abstract])) OR (Health Correlates[Title/Abstract])) OR (Correlates, Health[Title/Abstract])) OR (Population at Risk[Title/Abstract])) OR (Populations at Risk[Title/Abstract])) OR (Risk Scores[Title/Abstract])) OR (Risk Score[Title/Abstract])) OR (Score, Risk[Title/Abstract])) OR (Risk Factor Scores[Title/Abstract])) OR (Risk Factor Score[Title/Abstract])) OR (Score, Risk Factor[Title/Abstract]))) AND (((((((((((((((((((((((("Acute Kidney Injury"[Mesh]) OR (Acute Kidney Injuries[Title/Abstract])) OR (Kidney Injuries, Acute[Title/Abstract])) OR (Kidney Injury, Acute[Title/Abstract])) OR (Acute Renal Injury[Title/Abstract])) OR (Acute Renal Injuries[Title/Abstract])) OR (Renal Injuries, Acute[Title/Abstract])) OR (Renal Injury, Acute[Title/Abstract])) OR (Kidney Failure, Acute[Title/Abstract])) OR (Acute Kidney Failures[Title/Abstract])) OR (Kidney Failures, Acute[Title/Abstract])) OR (Acute Renal Failure[Title/Abstract])) OR (Acute Renal Failures[Title/Abstract])) OR (Renal Failures, Acute[Title/Abstract])) OR (Renal Failure, Acute[Title/Abstract])) OR (Acute Kidney Failure[Title/Abstract])) OR (Renal Insufficiency, Acute[Title/Abstract])) OR (Acute Renal Insufficiencies[Title/Abstract])) OR (Renal Insufficiencies, Acute[Title/Abstract])) OR (Acute Renal Insufficiency[Title/Abstract])) OR (Kidney Insufficiency, Acute[Title/Abstract])) OR (Acute Kidney Insufficiencies[Title/Abstract])) OR (Kidney Insufficiencies, Acute[Title/Abstract])) OR (Acute Kidney Insufficiency[Title/Abstract]))

**Cochrane**

#12 MeSH descriptor: [Immune Checkpoint Inhibitors] explode all trees 93

#13 (Immune Checkpoint Inhibitors or Checkpoint Inhibitors, Immune or Immune Checkpoint Inhibitor or Checkpoint Inhibitor, Immune or Immune Checkpoint Blockers or Checkpoint Blockers, Immune or Immune Checkpoint Blockade or Checkpoint Blockade, Immune or Immune Checkpoint Inhibition or Checkpoint Inhibition, Immune or PD L1 Inhibitors or PD L1 Inhibitors or PD L1 Inhibitor or PD L1 Inhibitor or Programmed Death Ligand 1 Inhibitors or Programmed Death Ligand 1 Inhibitors or PD 1 PD L1 Blockade or Blockade, PD 1 PD L1 or PD 1 PD L1 Blockade or CTLA 4 Inhibitors or CTLA 4 Inhibitors or CTLA 4 Inhibitor or CTLA 4 Inhibitor or Cytotoxic T Lymphocyte Associated Protein 4 Inhibitors or Cytotoxic T Lymphocyte Associated Protein 4 Inhibitors or Cytotoxic T Lymphocyte Associated Protein 4 Inhibitor or Cytotoxic T Lymphocyte Associated Protein 4 Inhibitor or PD 1 Inhibitors or PD 1 Inhibitors or PD 1 Inhibitor or Inhibitor, PD 1 or PD 1 Inhibitor or Programmed Cell Death Protein 1 Inhibitor or Programmed Cell Death Protein 1 Inhibitors):ti,ab,kw (Word variations have been searched) 7219

#14 #12 or #13 7219

#15 MeSH descriptor: [Risk Factors] explode all trees 26414

#16 (Factor, Risk or Risk Factor or Social Risk Factors or Factor, Social Risk or Factors, Social Risk or Risk Factor, Social or Risk Factors, Social or Social Risk Factor or Health Correlates or Correlates, Health or Population at Risk or Populations at Risk or Risk Scores or Risk Score or Score, Risk or Risk Factor Scores or Risk Factor Score or Score, Risk Factor):ti,ab,kw (Word variations have been searched) 174520

#17 #15 or #16 174520

#18 #14 and #17 802

#19 MeSH descriptor: [Acute Kidney Injury] explode all trees 1699

#20 (Acute Kidney Injuries or Kidney Injuries, Acute or Kidney Injury, Acute or Acute Renal Injury or Acute Renal Injuries or Renal Injuries, Acute or Renal Injury, Acute or Kidney Failure, Acute or Acute Kidney Failures or Kidney Failures, Acute or Acute Renal Failure or Acute Renal Failures or Renal Failures, Acute or Renal Failure, Acute or Acute Kidney Failure or Renal Insufficiency, Acute or Acute Renal Insufficiencies or Renal Insufficiencies, Acute or Acute Renal Insufficiency or Kidney Insufficiency, Acute or Acute Kidney Insufficiencies or Kidney Insufficiencies, Acute or Acute Kidney Insufficiency):ti,ab,kw (Word variations have been searched) 10975

#21 #19 or #20 10996

#22 #18 and #21 21

**Web of science**

(TS=(Acute Kidney Injury) OR AB=(AKI OR Acute Kidney Injuries OR Kidney Injuries, Acute OR Kidney Injury, Acute OR Acute Renal Injury OR Acute Renal Injuries OR Renal Injuries, Acute OR Renal Injury, Acute OR Kidney Failure, Acute OR Acute Kidney Failures OR Kidney Failures, Acute OR Acute Renal Failure OR Acute Renal Failures OR Renal Failures, Acute OR Renal Failure, Acute OR Acute Kidney Failure OR Renal Insufficiency, Acute OR Acute Renal Insufficiencies OR Renal Insufficiencies, Acute OR Acute Renal Insufficiency OR Kidney Insufficiency, Acute OR Acute Kidney Insufficiencies OR Kidney Insufficiencies, Acute OR Acute Kidney Insufficiency)) AND (TS=(Immune Checkpoint Inhibitors ) OR AB=(Checkpoint Inhibitors, Immune OR Immune Checkpoint Inhibitor OR Checkpoint Inhibitor, Immune OR Immune Checkpoint Blockers OR Checkpoint Blockers, Immune OR Immune Checkpoint Blockade OR Checkpoint Blockade, Immune OR Immune Checkpoint Inhibition OR Checkpoint Inhibition, Immune OR PD-L1 Inhibitors OR PD L1 Inhibitors OR PD-L1 Inhibitor OR PD L1 Inhibitor OR Programmed Death-Ligand 1 Inhibitors OR Programmed Death Ligand 1 Inhibitors OR PD-1-PD-L1 Blockade OR Blockade, PD-1-PD-L1 OR PD 1 PD L1 Blockade OR CTLA-4 Inhibitors OR CTLA 4 Inhibitors OR CTLA-4 Inhibitor OR CTLA 4 Inhibitor OR Cytotoxic T-Lymphocyte-Associated Protein 4 Inhibitors OR Cytotoxic T Lymphocyte Associated Protein 4 Inhibitors OR Cytotoxic T-Lymphocyte-Associated Protein 4 Inhibitor OR Cytotoxic T Lymphocyte Associated Protein 4 Inhibitor OR PD-1 Inhibitors OR PD 1 Inhibitors OR PD-1 Inhibitor OR Inhibitor, PD-1 OR PD 1 Inhibitor OR Programmed Cell Death Protein 1 Inhibitor OR Programmed Cell Death Protein 1 Inhibitors)) AND (TS=(Risk Factors) OR AB=(Factor, Risk OR Risk Factor OR Social Risk Factors OR Factor, Social Risk OR Factors, Social Risk OR Risk Factor, Social OR Risk Factors, Social OR Social Risk Factor OR Health Correlates OR Correlates, Health OR Population at Risk OR Populations at Risk OR Risk Scores OR Risk Score OR Score, Risk OR Risk Factor Scores OR Risk Factor Score OR Score, Risk Factor))

**Embase**

#10. #3 AND #6 AND #9 48

#9. #7 OR #8 1,467,000

#8. 'factor, risk':ab,ti OR 'risk factor':ab,ti OR 435,563

'social risk factors':ab,ti OR 'factor, social

risk':ab,ti OR 'factors, social risk':ab,ti OR

'risk factor, social':ab,ti OR 'risk factors,

social':ab,ti OR 'social risk factor':ab,ti OR

'health correlates':ab,ti OR 'correlates,

health':ab,ti OR 'population at risk':ab,ti OR

'populations at risk':ab,ti OR 'risk

scores':ab,ti OR 'risk score':ab,ti OR 'score,

risk':ab,ti OR 'risk factor scores':ab,ti OR

'risk factor score':ab,ti OR 'score, risk

factor':ab,ti

#7. 'risk factor'/exp 1,270,993

#6. #4 OR #5 37,832 19 Dec 2022

#5. 'checkpoint inhibitors, immune':ab,ti OR 'immune 25,647 checkpoint inhibitor':ab,ti OR 'checkpoint

inhibitor, immune':ab,ti OR 'immune checkpoint

blockers':ab,ti OR 'checkpoint blockers,

immune':ab,ti OR 'immune checkpoint

blockade':ab,ti OR 'checkpoint blockade,

immune':ab,ti OR 'immune checkpoint

inhibition':ab,ti OR 'checkpoint inhibition,

immune':ab,ti OR 'pd-l1 inhibitors':ab,ti OR 'pd

l1 inhibitors':ab,ti OR 'pd-l1 inhibitor':ab,ti

OR 'pd l1 inhibitor':ab,ti OR 'programmed

death-ligand 1 inhibitors':ab,ti OR 'programmed

death ligand 1 inhibitors':ab,ti OR 'pd-1-pd-l1

blockade':ab,ti OR 'blockade, pd-1-pd-l1':ab,ti

OR 'pd 1 pd l1 blockade':ab,ti OR 'ctla-4

inhibitors':ab,ti OR 'ctla 4 inhibitors':ab,ti OR

'ctla-4 inhibitor':ab,ti OR 'ctla 4

inhibitor':ab,ti OR 'cytotoxic

t-lymphocyte-associated protein 4

inhibitors':ab,ti OR 'cytotoxic t lymphocyte

associated protein 4 inhibitors':ab,ti OR

'cytotoxic t-lymphocyte-associated protein 4

inhibitor':ab,ti OR 'cytotoxic t lymphocyte

associated protein 4 inhibitor':ab,ti OR 'pd-1

inhibitors':ab,ti OR 'pd 1 inhibitors':ab,ti OR

'pd-1 inhibitor':ab,ti OR 'inhibitor, pd-1':ab,ti

OR 'pd 1 inhibitor':ab,ti OR 'programmed cell

death protein 1 inhibitor':ab,ti OR 'programmed

cell death protein 1 inhibitors':ab,ti

#4. 'immune checkpoint inhibitor'/exp 17,834

#3. #1 OR #2 135,151

#2. 'acute kidney failure'/exp 118,643

#1. aki:ab,ti OR 'acute kidney injuries':ab,ti OR 74,573

'kidney injuries, acute':ab,ti OR 'kidney injury,

acute':ab,ti OR 'acute renal injury':ab,ti OR

'acute renal injuries':ab,ti OR 'renal injuries,

acute':ab,ti OR 'renal injury, acute':ab,ti OR

'kidney failure, acute':ab,ti OR 'acute kidney

failures':ab,ti OR 'kidney failures, acute':ab,ti

OR 'acute renal failure':ab,ti OR 'acute renal

failures':ab,ti OR 'renal failures, acute':ab,ti

OR 'renal failure, acute':ab,ti OR 'acute kidney

failure':ab,ti OR 'renal insufficiency,

acute':ab,ti OR 'acute renal

insufficiencies':ab,ti OR 'renal insufficiencies,

acute':ab,ti OR 'acute renal insufficiency':ab,ti

OR 'kidney insufficiency, acute':ab,ti OR 'acute

kidney insufficiencies':ab,ti OR 'kidney

insufficiencies, acute':ab,ti OR 'acute kidney

insufficiency':ab,ti
