# Supplementary material for: Incidence and risk factors of acute kidney injury in cancer patients treated with immune checkpoint inhibitors: a systematic review and meta-analysis
Source: Front Immunol. 2023 May 29;14:1173952. doi: 10.3389/fimmu.2023.1173952 (PMC10258324; doi:10.3389/fimmu.2023.1173952)
Supplement: Supplementary file 2 [file DataSheet_2.doc]

Supplementary Material

**SUPPLEMENTARY APPENDIX**

**Appendix S1.** Search strategy for incidence

**Appendix S2.** Search strategy for risk factors

**Table S1**. Quality assessment of cohort and case-control studies using the Newcastle-Ottawa scale

**Table S2.** Sensitivity analysis for risk factors: age and PPI use

**Table S3.** Begg’s and Egger’s test for publication bias.

**Table S4.** The summary of other risk factors in included studies.

**Figure S1.** Risk of bias graph

**Figure S2.** Risk of bias summary

**Figure S3.** Forest plots of the odds ratio for gender (male)

**Figure S4.** Forest plots of the odds ratio for hypertension

**Figure S5.** Forest plots of the odds ratio for baseline eGFR

**Figure S6.** Forest plots of the odds ratio for pembrolizumab

**Figure S7.** Forest plots of the odds ratio for duration of ICIs

**Figure S8.** Forest plot of time from ICIs initiation to ICI-AKI

**Table S1**. Quality assessment of cohort and case-control studies using the Newcastle-Ottawa scale

| Study | Selection | | | | Comparability | Outcome | | | Quality score |
| --- | --- | --- | --- | --- | --- | --- | --- | --- | --- |
|  | Representativeness of the exposed cohort | Selection of the non exposed cohort | Ascertainment of exposure | Demonstration that outcome of interest was not present at the start of the design or analysis | Comparability of cohorts on the biasis of the design or analysis | Assesement of outcome | Was follow-up long enough for outcomes to occure | Adequacy of follow up of cohorts |  |
| Abdelrahim,2021 | 1 | 1 | 1 | 1 | 2 | 1 | 0 | 1 | 8 |
| Cortazar,2020 | 1 | 1 | 1 | 1 | 2 | 1 | 0 | 1 | 8 |
| De Giglio,2022 | 1 | 1 | 1 | 1 | 2 | 1 | 0 | 1 | 8 |
| Espi,2021 | 1 | 1 | 1 | 1 | 2 | 1 | 0 | 1 | 8 |
| Garcia-Carro,2022 | 1 | 1 | 1 | 1 | 2 | 1 | 1 | 1 | 9 |
| Gerard,2022 | 1 | 1 | 0 | 1 | 2 | 1 | 0 | 0 | 6 |
| Gupta,2021 | 1 | 1 | 1 | 1 | 2 | 1 | 0 | 0 | 7 |
| Gupta,2022 | 1 | 1 | 1 | 1 | 2 | 1 | 0 | 0 | 7 |
| Isik,2021 | 1 | 1 | 1 | 1 | 2 | 1 | 0 | 1 | 8 |
| Ji,2022 | 0 | 1 | 1 | 1 | 2 | 1 | 1 | 0 | 7 |
| Koks,2021 | 0 | 1 | 1 | 1 | 2 | 1 | 0 | 1 | 7 |
| Liu,2022 | 0 | 1 | 1 | 1 | 2 | 1 | 0 | 1 | 7 |
| Meraz-Muñoz,2020 | 1 | 1 | 1 | 1 | 2 | 1 | 0 | 1 | 8 |
| Qin,2022 | 0 | 1 | 1 | 1 | 2 | 1 | 0 | 1 | 7 |
| Seethapathy,2019 | 0 | 1 | 1 | 1 | 2 | 1 | 0 | 0 | 6 |
| Seethapathy,2020 | 1 | 1 | 1 | 1 | 2 | 1 | 0 | 1 | 8 |
| Seethapathy,2021 | 1 | 1 | 1 | 1 | 2 | 1 | 0 | 0 | 7 |
| Shimamura,2021 | 0 | 1 | 1 | 1 | 2 | 1 | 0 | 1 | 7 |
| Sorah,2021 | 0 | 1 | 1 | 1 | 2 | 1 | 0 | 0 | 6 |
| Stein,2021 | 0 | 1 | 1 | 1 | 2 | 1 | 1 | 1 | 8 |
| Strohbehn，2021 | 1 | 0 | 1 | 1 | 2 | 1 | 0 | 0 | 6 |
| Trevisani,2022 | 1 | 1 | 1 | 1 | 2 | 1 | 0 | 0 | 7 |
| Yu,2022 | 1 | 1 | 1 | 1 | 2 | 1 | 0 | 1 | 8 |

**Table S2.** Sensitivity analysis for risk factors: age and PPI use

| Study omitted | OR(95%CI) |
| --- | --- |
| Age | 1.01(1.00,1.03) |
| Cortazar,2020 | 1.01(1.00,1.03) |
| Espi,2021 | 1.01(1.00,1.02) |
| Gerard,2022 | 1.01(1.00,1.03) |
| Gupta,2021 | 1.01(1.00,1.03) |
| Gupta,2022 | 1.02(1.01,1.04) |
| Koks,2021 | 1.01(1.00,1.03) |
| Liu,2022 | 1.02(1.00,1.03) |
| Qin,2022 | 1.01(1.00,1.02) |
| Shimamura,2021 | 1.01(1.00,1.03) |
|  |  |
| PPI | 2.23(1.88,2.64) |
| Abdelrahim,2021 | 2.22(1.86,2.65) |
| Cortazar,2020 | 2.14(1.78,2.57) |
| Gerard,2022 | 2.24(1.86,2.69) |
| Gupta,2021 | 2.15(1.75,2.65) |
| Ji,2022 | 2.25(1.88,2.68) |
| Koks,2021 | 2.32(1.93,2.78) |
| Qin,2022 | 2.25(1.90,2.67) |
| Stein,2021 | 2.26(1.89,2.69) |

**Table S3.** Begg’s test and Eegger’s test for publication bias.

| Variables | Begg’s test | Egger’s test |
| --- | --- | --- |
| Age | 0.917 | 0.936 |
| PPI | 0.174 | 0.141 |

**Table S4.** The summary of other risk factors in included studies.

| Study | Factors | Effect size (95% CI) |
| --- | --- | --- |
| Abdelrahim,2021 | Asian(compared to White) | 4.18(1.09,16.04) |
| Cortazar,2020 | Gender(female) | 1.05(0.67,1.65) |
|  | Prior autoimmune disease | 1.08(0.55,2.11) |
| Gerard,2022 | Body mass index | 0.75(0.26,2.18) |
|  | Renal cancer | 1.42(0.67,3.00) |
|  | Hypouricemic | 1.81(0.66,4.99) |
| Gupta,2022 | Coronary artery disease | 1.44(1.03,2.02) |
|  | Triplet therapy(vs. monotherapy) | 1.19(0.88,1.61) |
| Ji,2022 | Anemia | 1.95(1.16,3.28) |
|  | Nivolumab | 1.20(0.78,1.87) |
|  | Toripalimab | 0.44(0.04,5.33) |
|  | Sintilimab | 0.53(0.27,1.03) |
|  | Antibiotics | 2.56(1.10,5.95) |
|  | Albumin |  |
|  | ＜30g/L | 1.62(1.17,2.23) |
|  | 30-35g/L | 0.58(0.32,1.09) |
| Koks,2021 | Gender(female) | 1.24(0.82,1.88) |
|  | Melanoma | 1 |
|  | Non small-cell lung cancer | 0.67(0.38,1.19) |
|  | Gynecologic cancer | 3.91(1.55,9.85) |
|  | Urinary tract cancer | 0.93(0.44,2.00) |
|  | Other cancer | 1.28(0.67,2.44) |
|  | Charlton Comorbidity Index |  |
|  | 0-3 | 1 |
|  | 4-7 | 1.27(0.36,4.56) |
|  | 8-11 | 1.67(0.44,6.32) |
|  | ≥12 | 1.37(0.56,17.97) |
|  | Prior chemotherapy or targeted therapy | 1.41(0.90,2.21) |
|  | Nivolumab | 1 |
|  | Other ICIs | 0.49(0.18,1.29) |
| Liu,2022 | First line treatment | 2.23(0.98,5.10) |
|  | Previous surgery | 0.37(0.08,1.71) |
| Qin,2022 | ICI cycle | 1.17(1.02,1.33) |
| Seethapathy,2019 | CTLA4 versus PD1 | 1.85(1.05,3.27) |
|  | PDL1 versus PD1 | 1.38(0.43,4.45) |
| Shimamura,2021 | Liver disease | 11.1(1.82,67.6) |
|  | Atezolizumab | 1.67(0.26,10.7) |
| Stein,2021 | Corticosteroids | 1.54(0.72,3.27) |


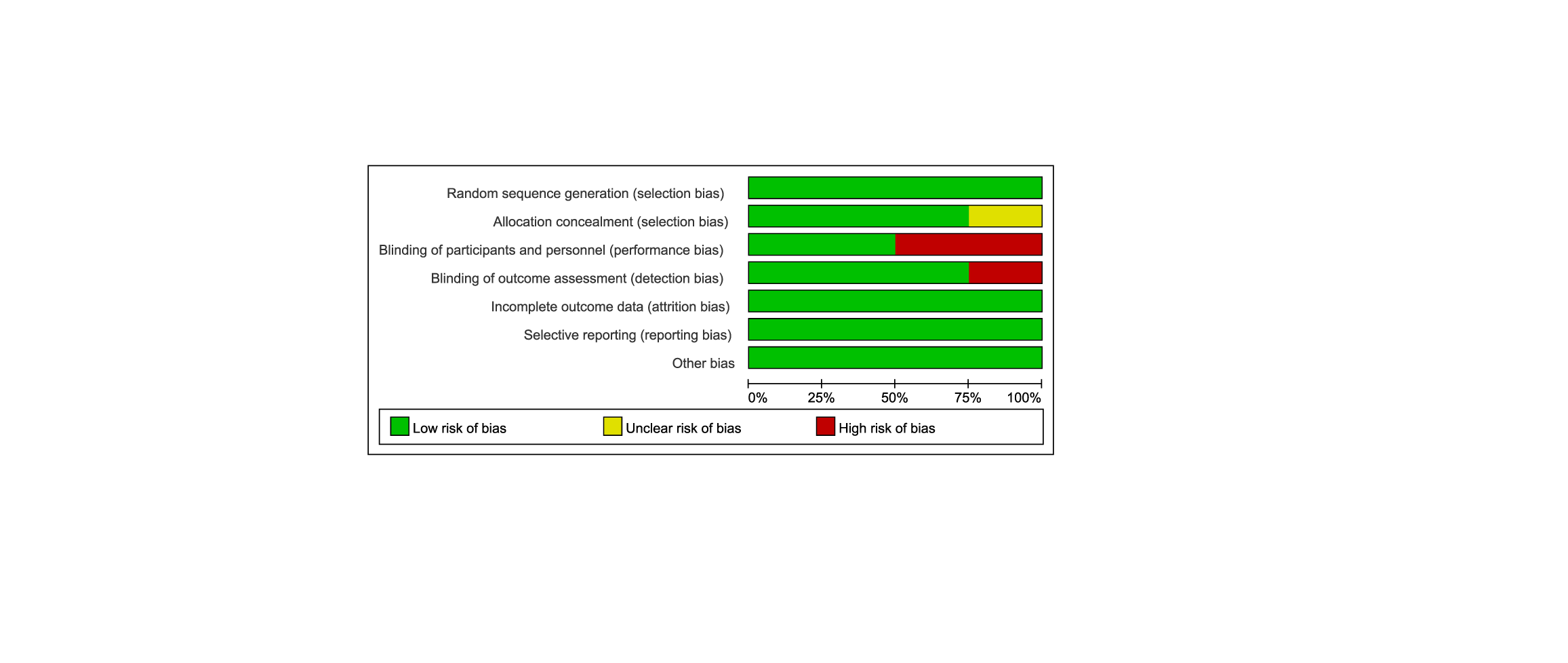


**Figure S1.** Risk of bias graph


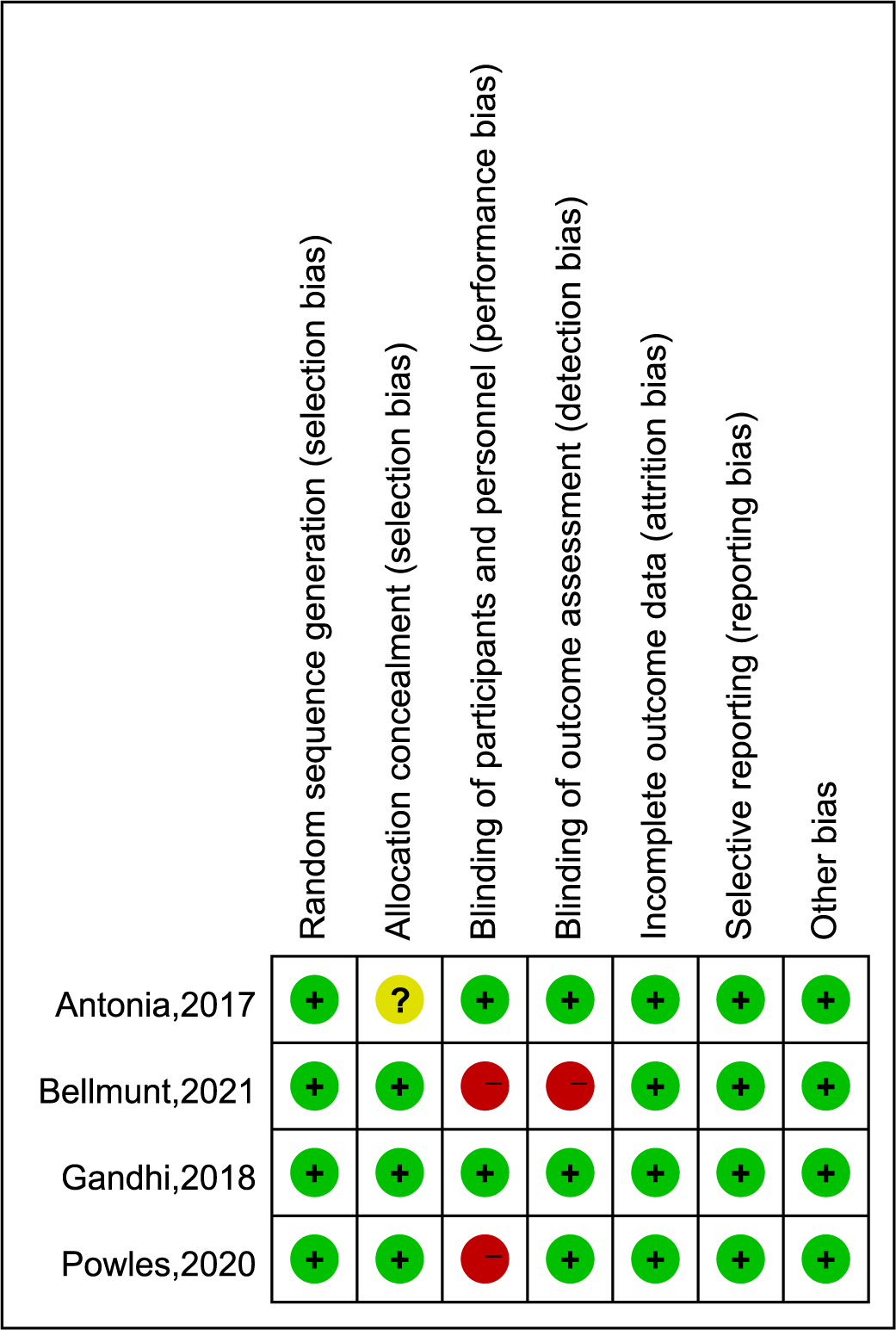


**Figure S2.** Risk of bias summary


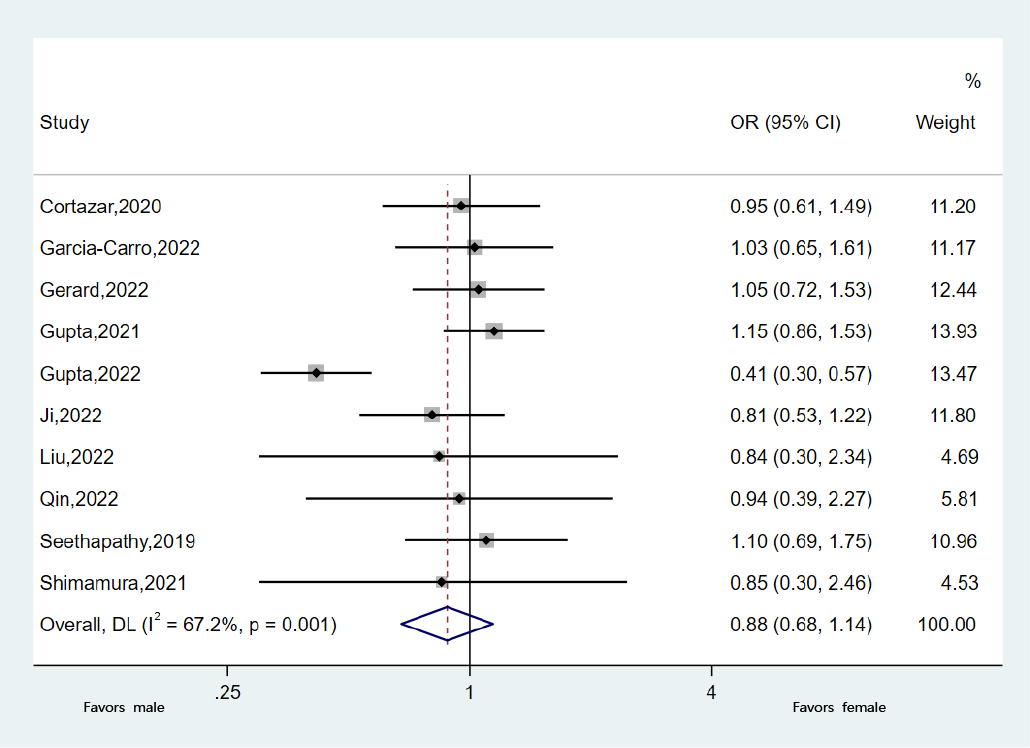


**Figure S3.** Forest plots of the odds ratio for gender (male)


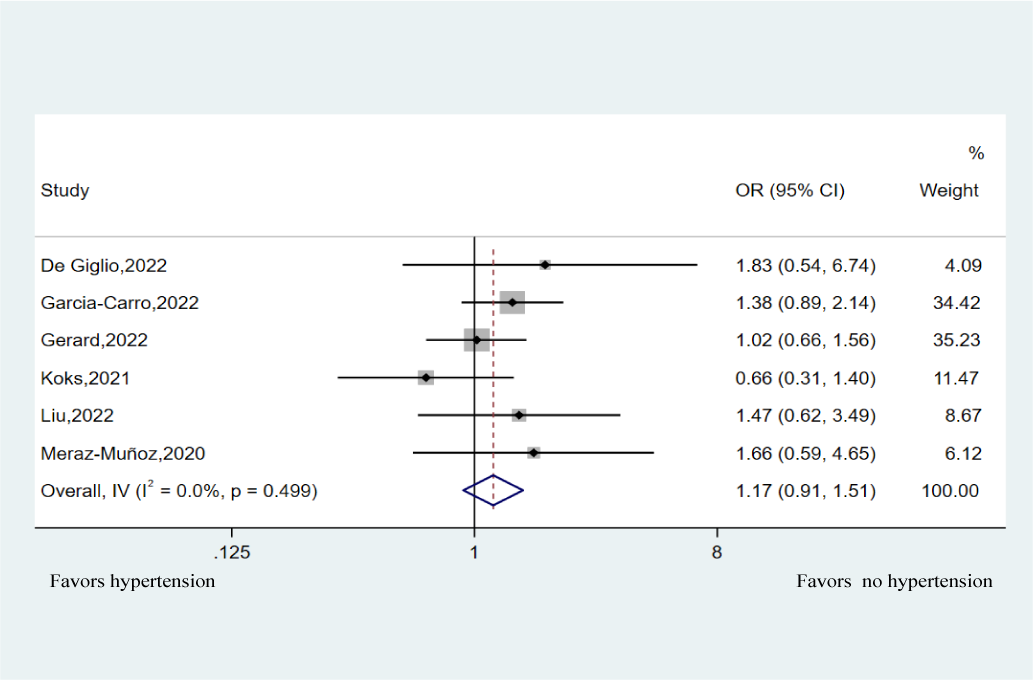


**Figure S4.** Forest plots of the odds ratio for hypertension


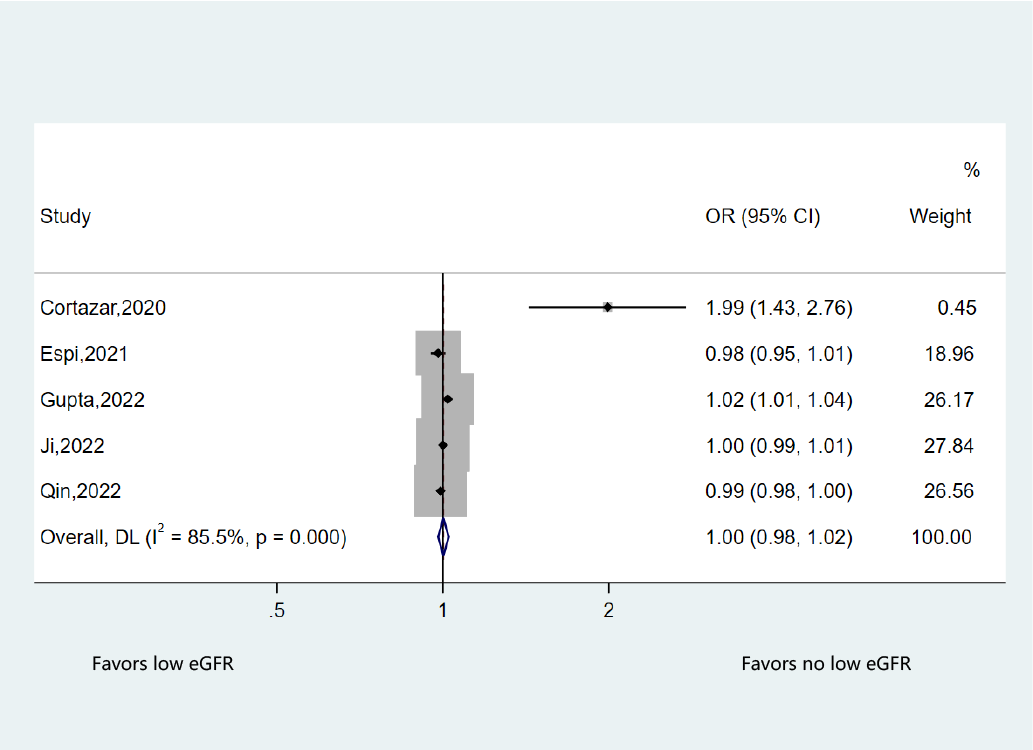


**Figure S5.** Forest plots of the odds ratio for baseline eGFR


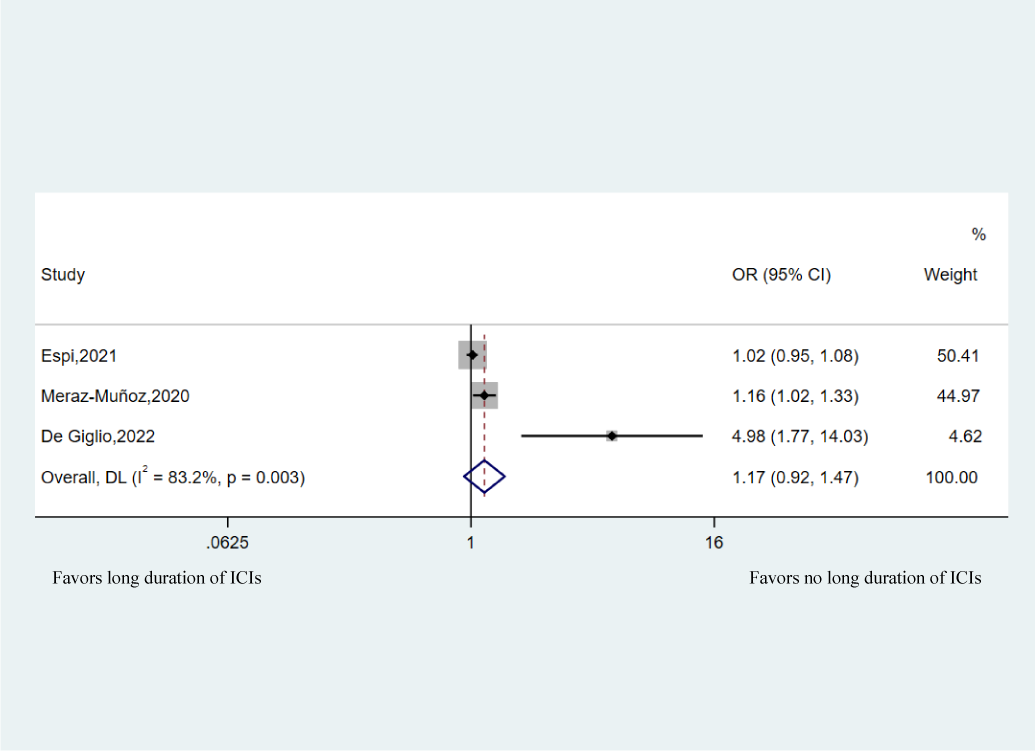


**Figure S6.** Forest plots of the odds ratio for pembrolizumab


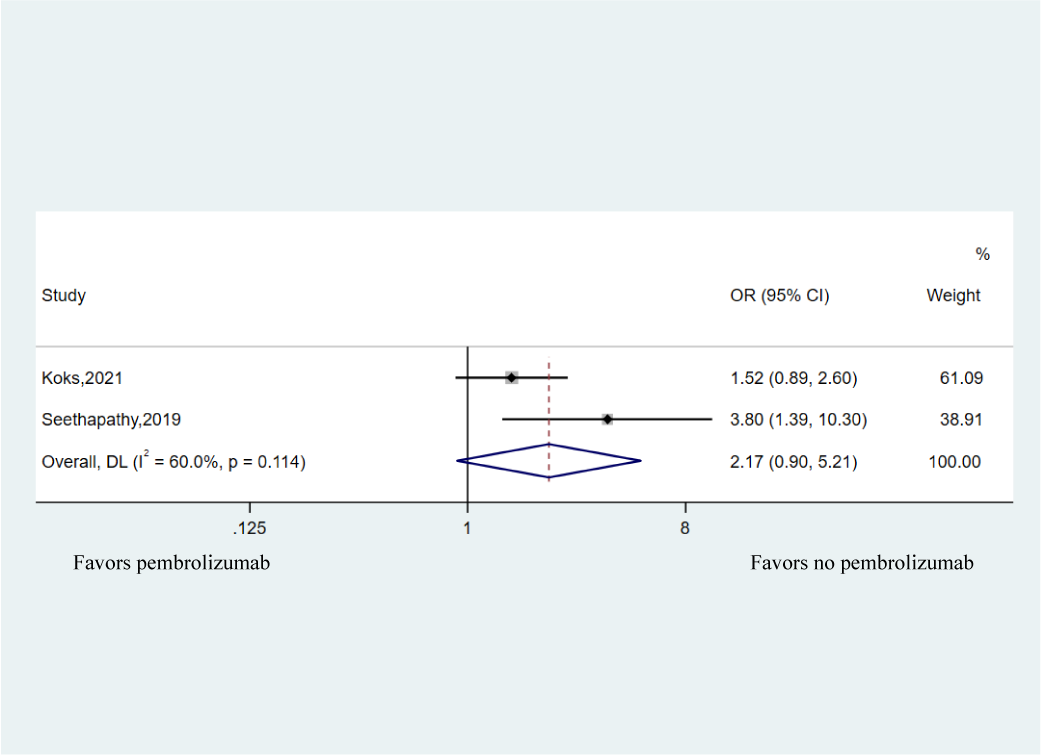


**Figure S7.** Forest plots of the odds ratio for duration of ICIs


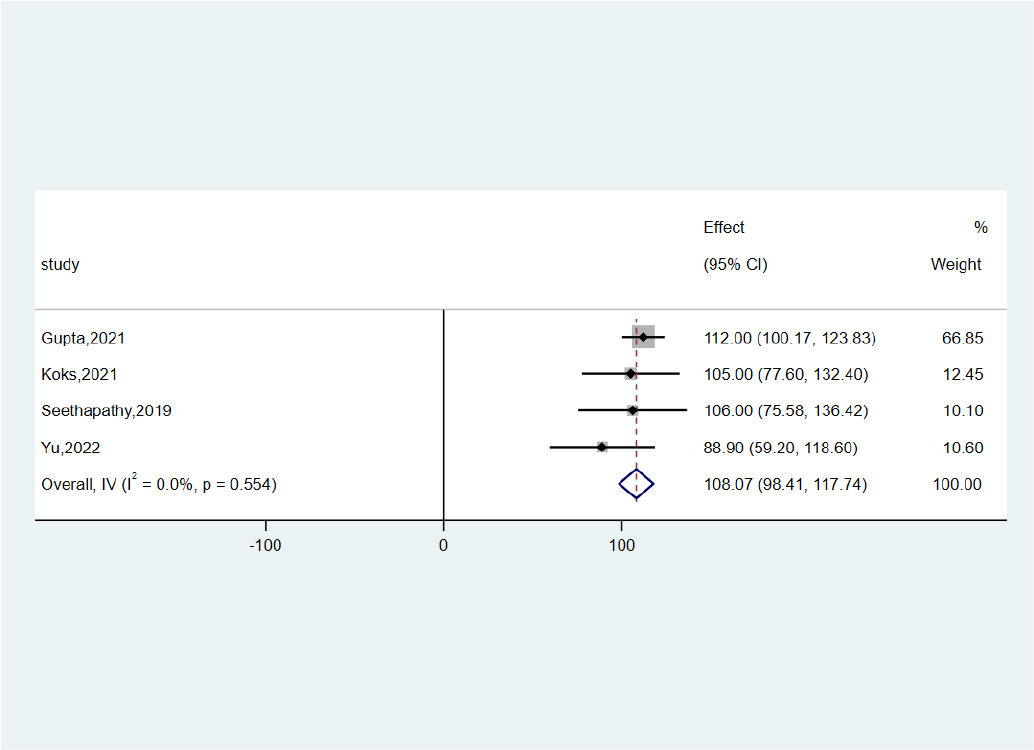


**Figure S8.** Forest plot of time from ICIs initiation to ICI-AKI
